# True, justified, belief? Partisanship weakens the positive effect of news media literacy on fake news detection

**DOI:** 10.3389/fpsyg.2023.1242865

**Published:** 2023-09-26

**Authors:** Daniel Jeffrey Sude, Gil Sharon, Shira Dvir-Gvirsman

**Affiliations:** ^1^Department of Organizational Sciences and Communication, George Washington University, Washington, DC, United States; ^2^DAN Department of Communication, Tel Aviv University, Tel Aviv, Israel

**Keywords:** fake news, misinformation, journalism, online news, social media, credibility

## Abstract

To investigate how people assess whether politically consistent news is real or fake, two studies (*N =* 1,008; *N =* 1,397) with adult American participants conducted in 2020 and 2022 utilized a within-subjects experimental design to investigate perceptions of news accuracy. When a mock Facebook post with either fake (Study 1) or real (Study 2) news content was attributed to an alternative (vs. a mainstream) news outlet, it was, on average, perceived to be less accurate. Those with beliefs reflecting News Media Literacy demonstrated greater sensitivity to the outlet’s status. This relationship was itself contingent on the strength of the participant’s partisan identity. Strong partisans high in News Media Literacy defended the accuracy of politically consistent content, even while recognizing that an outlet was unfamiliar. These results highlight the fundamental importance of looking at the interaction between user-traits and features of social media news posts when examining learning from political news on social media.

## Introduction

From the beginning of recorded history, we have records of lies and misinformation (Burkhardt, 2017). In recent years, it seems that the societal consequences of misinformation—from attempts to overturn the 2020 U.S. presidential election to active resistance to efforts to counter COVID-19 (Hornik et al., 2021; Montagni et al., 2021)—have become more disastrous. This intensification can be partly attributed to the participatory culture of Web 2.0, which facilitates unprecedented virality. In choosing what to share on social media, everyday users occupy a central and sometimes destructive place in the information environment (Lee and Shin, 2021).

Emphasizing users’ agency, a fundamental question is why individuals spread misinformation. Answering this question requires pinpointing factors that impact identifying misinformation in the first place (e.g., Tandoc et al., 2018; Edgerly et al., 2020; Waruwu et al., 2021). This question is of relevance to one form of misinformation known as “fake news:” inaccurate and purposefully misleading content that uses the same format and appearance as legitimate news sources (Allcott and Gentzkow, 2017; Lazer et al., 2018; Tandoc et al., 2018; Waisbord, 2018). Since fake news is disguised as legitimate news, identifying its untruthfulness can be difficult.

Building on research from the credibility and news media literacy perspectives, the current studies identify user-level and social media post-level factors that impact accuracy judgments. These studies were conducted to identify protective factors that limit the spread of fake news, but simultaneously they highlight the limitations of these factors. In doing so, they point the way forward for the design of novel interventions.

To develop its perspective, the current analysis first reviews key theoretical perspectives on why people share fake news on social media. It builds upon Pennycook and Rand’s (2021) model, which distinguishes inattention-based, preference-based, and confusion-based sharing of fake news. Hypotheses derived were then tested over the course of two studies, each utilizing a large sample of adult Americans, *via* within-subjects experimental designs manipulating the ostensible news outlet of social media posts. Study 1 examined fake news headlines, drawn from real world source of fake news. Study 2 sought to replicate and extend findings from Study 1, utilizing real news headlines and further collecting impressions of the outlets, generally.

### Fake news: a social (media) problem

The modern news landscape contains a mélange of content from mainstream news outlets, citizen journalists (Wall, 2018), and alternative news outlets (Waisbord, 2018). Alternative news outlets are a concern due to lower reporting standards and, at times, acts of deliberate deception (e.g., Lazer et al., 2018; Marwick and Lewis, 2018; Tandoc et al., 2018). Between 2016 and 2020, engagement with all news sites (Altay et al., 2020), including fake and alternative sites (Rogers, 2020), increased. While a relatively small number of users share fake news (e.g., Guess et al., 2019), these posts can garner a substantial amount of user engagement, which increases their visibility (e.g., Newberry, 2022).

Because social media users have a fundamental role in news dissemination (Lee and Shin, 2021), many scholars take a user-centered approach, asking how users infer that content is ‘shareworthy’. Research hitherto can be roughly divided into two groups: one investigating content and textual elements that contribute to virality, one investigating the identity and motivations of users sharing fake news.

While these lines of research are highly productive, the current analysis embraces a different approach. Specifically, this analysis focuses on the interaction between the news outlet as a source cue and the user’s news media literacy. The underlying assumption is that any decision to engage with news results from the “right match” between a given news story and a given user (Hopp et al., 2020). Thus, studies should not investigate what type of fake news stories are being shared or who shares them, as if these are two unrelated questions. Instead, they should be investigating the interaction between these factors.

To demonstrate the possible benefits of this approach, we focus on what may at first glance appear to be a ‘sharing paradox’. While generally users are aware that sharing fake news might harm their reputation (Altay et al., 2020), “social media sharing judgments are actually quite divergent from judgments about accuracy … many people were apparently willing to share content that they could have identified as being inaccurate” (Pennycook and Rand, 2021, p. 393). This behavior can sometimes be the result of lack of attention: The “*inattention-based account* argues that people have a strong preference to only share accurate content but that the social media context distracts them from this preference” (Pennycook and Rand, 2021, p. 395). Social media can make it less likely that users are motivated and able to assess whether information is accurate, due to a range of features including interfaces that emphasize social information over relevant news cues (Dvir-Gvirsman, 2019; Kalogeropoulos et al., 2019) and “information context collapse” – a complex array of different genres of information intermixed in the same feed which can hinder deliberative processing (Pearson, 2021).

At the same time, even under ideal conditions, as when users are explicitly reminded to consider whether information is accurate (Fazio, 2020; Pennycook and Rand, 2021), some individual users may be more motivated than others to pay attention to relevant contextual cues. A growing body of research, discussed in more detail below, demonstrates that relevant literacies may both provide people with the tools to navigate the sometimes overwhelming complexity of online spaces (e.g., Jones-Jang et al., 2021) and at the same time motivate them to do so (e.g., Tully et al., 2020). These users should simultaneously consider whether content is accurate and be less prone to error, as they pay attention to relevant cues.

However, the motivation to be accurate is further subject to a number of biases (see Sude and Knobloch-Westerwick, 2022, for a review). Further, even when people are aware of the potential for bias, they may encounter difficulties correcting for it (Wegener and Petty, 1997; Chien et al., 2014). Thus, even individuals who are motivated to engage in thoughtful processing may still embrace news content from a suspicious or lower quality source if they are biased in its favor. This circumstance is the object of the current analysis.

In the next two sections, we argue that even under the following ideal conditions—all users are prompted to consider the accuracy of news headlines, the content is clearly attributed to popular (CNN, Fox, ABC) vs. alternative (Just The News) outlets, and users are concerned with bias (high News Media Literacy users)— the intensity of a users’ political commitments will still lead to disregarding relevant cues and thus increase the likelihood of erroneously believing fake news. See the discussion for suggested interventions targeting users whose intense political commitments may make them more susceptible to adopting erroneous beliefs.

## The role of outlet cues: promoting confidence or caution

Many factors could impact perceived recognition of news content, including consonance with users’ existing beliefs (Tappin et al., 2021), level of topic-specific knowledge, as well, targeted in the current experiments, whether the news outlet itself is recognized by the user. This latter variable is particularly important, as, in the absence of prior content knowledge, it is reasonable for users to be more skeptical of information from unfamiliar sources. It behooves us to ask the following questions: (1) Which users attend to whether an outlet is alternative versus mainstream? and (2) When do users perceive content as accurate, despite not recognizing its associated outlet?

The extant empirical work suggests that users often indeed realize that they do not recognize an outlet, and in turn perceive its content to be less accurate. Mainstream news outlets are often rated as more trustworthy than either fake or hyperpartisan outlets, accounting for partisan bias (Pennycook and Rand, 2019). The credibility of even real news headlines can diminish when attributed to alternative news outlets (Dias et al., 2020). This observed credibility advantage of mainstream news outlets is partly driven by branding. Importantly, news brand recognition impacts sharing via an increase in perceived credibility (Bobkowski, 2015; Kümpel et al., 2015; Rudat and Buder, 2015). Further, brand recognition can have a strong, direct, effect on sharing likelihood, at least when users do not report personal involvement with the topic (Nekmat, 2020).

However, the relationship between the credibility of news content and perceived credibility of news outlets can be quite complex and even bi-directional (Collins et al., 2018). The credibility of content can also influence the credibility of sources (e.g., Slater and Rouner, 1996; Collins et al., 2018). The relationship between source and content credibility may depend on which cue is deemed the most useful. Impacts of content can be particularly strong when a source has no brand reputation (Altay et al., 2020; Johnson et al., 2020). Impacts of source credibility may be particularly strong when content itself is unfamiliar (Lucassen and Schraagen, 2013). However, the overarching pattern, in summary, is that many users pay attention to whether they recognize a news outlet and take this level of recognition into account when assessing the credibility of its content.

The central contention of the current analysis is that the previously observed empirical pattern, in which sources or content that are not perceived to be recognizable are in turn treated with caution, is normatively desirable. The current analysis argues that in age of the proliferation of smaller, alternative, and hyper-partisan news outlets, if there is insufficient information to assess the credibility of the content, because either source or content are perceived to be unfamiliar, users are wise to be cautious, rather than open-minded.

Given these findings, Study 1 attributed partisan-consonant fake news content to different outlets in a within subjects design. Consonant fake news content collected from real world sources of fake news was attributed in mock Facebook posts to both mainstream liberal (CNN) and conservative (Fox) news outlet brands, as well as to an alternative news outlet brand (Just The News). Note that even outlets with partisan associations can be considered mainstream (Shearer and Mitchell, 2021).

Study 2 replicated and extended Study 1, utilizing *real* news headlines and an additional, mainstream but not strongly partisan, news outlet, ABC News. Both studies assessed participants’ impressions of these posts in terms of perceived (a) recognition and (b) accuracy. To clarify findings in Study 1, in Study 2, participants were additionally asked about impressions of the outlets themselves, independent of any particular content, in order to differentiate perceptions of the outlet from perceptions of the content and, in particular, to demonstrate that the alternative outlet (Just The News) itself was indeed less recognizable.

This alternative news outlet brand was closely based on a real-world news outlet, “Just the News,” with which most US participants were predicted to be, on average, unfamiliar. Note that this proposition was upheld, at least per this sample, when tested in Study 2. Of note, the real-world outlet Just the News aligns with the definition of an alternative news outlet offered by Holt et al. (2019): Its tagline “Just News, No Noise” and its very title may be read as positioning “Just the News” in contrast to mainstream news outlets.

### A normative perspective on recognizability and credibility

While, from a normative perspective, the current analysis argues that it is good for users to be cautious of content from unfamiliar outlets, we acknowledge that mainstream news can certainly introduce misinformation and even disinformation. However, due to competition among news outlets to present the “right” perspective, other mainstream news outlets are likely to notice and seek to correct these “errors,” sometimes leading to a convergence on a common “truth” between even papers with opposing ideologies (Gentzkow and Shapiro, 2008).

Insulated from the attention of larger mainstream outlets, however, alternative news outlets are less likely to be subject to these corrective forces. Of course, alternative outlets can still defend their credibility by providing strong evidence in favor of their coverage. They can live up to their branding and provide valuable information to their audience. However, because they could also produce fraudulent information that goes undetected, readers should initially be skeptical. Simply put, until the reader investigates further, that reader should be inherently skeptical of content from an unfamiliar, unrecognized, outlet, treating it with caution rather than an open-minded neutrality.

### Perceived recognition versus perceived accuracy

The complex meta-cognitive process that generates the feeling of recognizing information is subject to many factors, beyond those easily manipulable in an experiment. For example, truly unfamiliar content (e.g., fake news content, on average) could be *falsely* recognized if it seems plausible or even merely similar to other information the user has encountered. In fact, even perceived recognition of real news content could be negatively impacted by its association with an unfamiliar source (see Dias et al., 2020, for related evidence).

This false recognition or lack thereof could influence processing fluency, a metacognitive cue that can in turn impact perceived accuracy (see Shulman and Bullock, 2019, for a review). Note, however, that perceived accuracy in turn reflects a variety of *other* factors, such as desirability or overall plausibility, that will be taken up again in the next section. *Ceteris paribus,* however, the following hypotheses are offered:

*H1*: Alternative (vs. mainstream) outlets are perceived to have general content that is (a) less recognizable and (b) less accurate.

*H2*: Specific partisan-consonant fake {real} news posts from alternative (vs. mainstream) outlets are perceived to be (a) less recognizable and (b) less accurate.

Importantly, while the preceding discussion emphasized each news outlet’s brand as a fundamentally important context cue, in line with our focus on the “match” between users and news posts, the next section reviews a key user-level variable, news media literacy, that we predicted would impact how users responded to the context cue.

## User-level factors: news media literacy and partisan bias

News media literacy (NML) acknowledges that publishers may not present the unvarnished truth but rather shape that truth in ways that can reflect a variety of biases, for example, bias toward negative news coverage or, of particular relevance here, bias in favor of a political perspective (Ashley et al., 2013). It is thus a key user-level factor that could shape attention to and interpretation of outlet cues.

The more general concept of media literacy is multidimensional. Potter (2004), for example, proposes a cognitive model in which complex knowledge structures—regarding media content, media industries, media effects, the world, and the self—interact with individual differences in epistemic habits to shape media literacy. From this perspective, in the right circumstances people will recognize incomplete and biased representations of information in the media. They may then seek out more complete and balanced perspectives.

Some news media literacy (NML) scales measure specific knowledge of news production processes as well as media effects (Maksl et al., 2015). General knowledge of news production processes may promote more caution with regards to evaluation of all news reporting, as all outlets have risk factors for incomplete or biased presentations of information: whether journalists are licensed, whether most outlets are profit-driven or not for profit, etc. Similar to information literacy (e.g., Boh Podgornik et al., 2016), measures of news media literacy per knowledge of objective factors can identify users who understand that news sources shape both the completeness and the bias of their content.

However, for political content in particular, the current analysis is focused on a more subjective user-level factor, awareness of bias. Ashley et al’s. (2013) scale as shortened by Jones-Jang et al. (2021) has this explicit focus, asking, for example, how much someone agrees that “The owner of a media company influences the media content” or that “Individuals can find news sources that reflect their own political value.” Perceptions of bias are both fundamentally important and, as discussed above, fundamentally subjective (Wegener and Petty, 1997; Chien et al., 2014).

We argue that greater awareness of the potential for bias drives higher NML users to actively consider the perspective of news producers, and thus notice whether a news outlet brand is recognized (mainstream) or unrecognized (alternative). Those higher in news media literacy believe that news outlets are active and potentially biased agents, shaping news coverage in order to achieve certain goals. *Ceteris paribus*, then, those higher in NML should ask themselves, what news outlet published this content and what were their goals?

*H3*: For an alternative news outlet (versus a mainstream news outlet), higher NML users perceive its general content to be (a) less recognizable and (b) less accurate.

*H4*: When specific partisan-consonant fake {real} news content is associated with an alternative news outlet (versus a mainstream news outlet), higher NML users perceive this content to be (a) less recognizable and (b) less accurate.

Further, however, we argue that mere awareness of the potential for bias is insufficient. While the role of partisan-bias in perceptions of news content is hotly debated (see Pennycook and Rand, 2021), it must be acknowledged that the mental pictures that people develop regarding news outlets can be quite biased, particularly along political dimensions (see Tully et al., 2020, for qualitative evidence). Perceptions of recognition could be subject to a motivated reasoning process (Kunda, 1990) or other psychological processes that contribute to biased credibility perceptions (for discussions see Druckman and McGrath, 2019; Sude and Knobloch-Westerwick, 2022). Notably, if recognizing content confers feelings that it is accurate, and perceiving that this content is accurate is desirable, then participants may be motivated to claim recognition.

In straightforward parallel, perceiving that partisan-favoring content is in fact accurate can be desirable, leading to partisan bias in accuracy perceptions. Politically-biased motivated reasoning is an extremely well-studied phenomenon (see Ditto et al., 2019 for a recent meta-analysis). From the current analyses’ perspective, however, it remains, empirically, on open question whether those high in NML will be as susceptible to bias.

For example, while those high in NML may as a rule be more skeptical of content from less recognized outlets, an outlet that delivers consonant political content, which may be deemed as both more desirable and more plausible, may instead gain a credibility advantage. While we have argued that people should be inherently skeptical of content from an alternative news outlet and hold it to an even higher standard of evidence, the average person may instead simply be neutral but open-minded regarding this content. The partisan-bias of that content could then sway the initially neutral impression to become a positive one.

At the same time, it is possible that NML will instead provoke a sober second thought, despite the user’s political commitments. To investigate the role of biased perceptions of bias, RQ1 is asked.

*RQ1*: Are impacts per (a) H3a, (b) H3b, (c) H4a, or (d) H4b further contingent on users’ partisan extremity?

## General methods

### Overview

Two studies were conducted, after obtaining Institutional Review Board approval at Tel Aviv University. After consenting, completing demographic measures, the news media literacy (NML) scale, and covariates, all participants saw several posts in random order, with each post representing an experimental treatment.

In Study 1, participants saw posts with politically-consonant *fake news* content, as well as distractor conditions including politically dissonant *fake news* and apolitical *fake news* content to obscure the purpose of the study. The outlet to which the content was attributed was varied (see Manipulation section).

Following each post, participants indicated whether they had seen or heard the story before (perceived recognition) and whether the story was accurate (perceived accuracy). They also reported their likelihood of sharing the content. Then, participants responded to a screening attention check item asking them to indicate the topic of the post they had just seen. As distractor items, they reported their emotional states. Closing the study, participants completed demographic measures related to their religious affiliation and education. Additionally, in Study 2, participants were asked not just about their impressions of these *posts* but about the *outlets* themselves. Study 2, thus, was designed to facilitate disentangling impressions of content from impressions of the outlets themselves.

### Participants

Potential participants were eligible to vote in US elections and reported that they were aware that they could be exposed to misinformation on the web. All participants were panel survey participants recruited by Qualtrics in return for pre-agreed upon rewards. Note that, prior to data collection, Qualtrics agreed to replace all participants who did not pass more than half of the screening items, asking us to disregard data from these individuals. For Study 1, participants were recruited in August of 2020: Data from 1,008 participants was retained. For Study 2, participants were recruited in February of 2022: Data from 1,397 participants was retained. As demonstrated in Table 1, across both studies, quota sampling ensured a diverse distribution in terms of gender, age, and region.

**Table 1:** Participant demographic characteristics.

Variable	Study 1 (*N* = 1,008)	Study 2 (*N* = 1,397)
*Age (%)*		
18–24	13.0	10.8
25–34	18.3	19.0
35–44	17.9	18.3
45–54	19.1	19.7
55–64	17.2	9.2
65 and older	14.5	22.9
*Gender (%)*		
Men	47.4	48.9
Women	51.2	51.1
*Education by highest degree (%)*		
Less than high school	1.3	2.2
High school	20.4	28.9
Some college or Associate’s degree	28.6	32.1
Bachelor’s	28.8	22.9
Master’s	17.0	11.5
Ph.D. or Professional Degree	4.1	2.4
*Region (%)*		
Midwest	21.5	19.0
Northeast	19.4	21.5
South	35.6	40.9
West	22.6	18.6
*Partisan affiliation (%)*		
Strong democrat	24.5	21.9
Not very strong democrat	11.4	10.4
Independent – Lean democrat	11.2	11.1
Independent	15.7	20.1
Independent – lean Republican	7.4	10.0
Not very strong Republican	9.1	9.1
Strong Republican	20.8	17.4
*Covariates*		
Political interest (1–7)	*M* = 4.55, *SD* = 1.28	*M* = 4.63, *SD* = 1.45
Trust in news media (1–7)	*M* = 3.51, *SD* = 1.62	*M* = 3.68, *SD* = 1.70
Trust in institutions (1–7)	*M* = 3.24, *SD* = 1.31	*M* = 4.00, *SD* = 1.36
Faith in intuition for facts (1–7)	*M* = 4.94, *SD* = 1.18	*M* = 5.05, *SD* = 1.13
Need for evidence (1–7)	*M* = 5.46, *SD* = 1.14	*M =* 5.54, *SD* = 1.09
Truth as Political (1–7)	*M* = 4.35, *SD* = 1.45	*M* = 4.34, *SD* = 1.47
Endorsement of Conspiracy theories (1–5)	*M* = 2.69, *SD* = 0.85	*M* = 2.57, *SD* = 0.88
News exposure (days of prior week, across types)	*M* = 3.69, *SD* = 1.63	*M* = 2.81, *SD* = 1.72
Information literacy	*M* = 1.71/5, *SD* = 1.12	*M = 1.76/5, SD* = 1.15

### Manipulation

Each post was in a Facebook format and consisted of a media brand (logo/picture, name of the news organization, and date), a title, an image, a URL of the news organization, and a headline. The stimulus posts are available upon request from the corresponding author. These examples cover every headline used. However, each headline was randomly assigned to an outlet, according to the respective study’s specifications as described below.

In both studies, participants viewed each post for a minimum of 9 s. In Study 1, one post presented consonant *fake news* content from CNN, from Fox News, and from an alternative brand (Just The News). To obscure the purpose of the study, distractor conditions presented other *fake news* posts attributed to ABC News, Natural News, and the DC Gazette. In Study 2, posts presented consonant *real news* content attributed to CNN, Fox News, ABC News (a mainstream and not highly partisan news site), and Just The News (an alternative news site).

For a detailed description of pretesting across both studies (see online Supplementary material Appendix A). As noted previously, all *fake news* posts represent headlines from real world articles sharing misinformation and all *real news* posts appeared in mainstream media outlets (also online Supplementary material Appendix B). Note that, to more closely resemble real social media posts on Facebook, which as a default contain a preview of the website, and typically contain an image, stimuli as pretested included images. While we cannot isolate the impact of the images chosen, pretesting at least establishes their parity along the desired dimensions. Importantly, posts studied here were all rated as favoring the participant’s political party. Thus, the stimuli were designed to balance ecological validity against a need for consistency. Note that the experimental manipulation applied only to the outlet cue itself, which was randomly assigned to the respective posts.

Last, regarding dissonance and consonance, The 15.7% of participants in Study 1 and the 20.1 of participants in Study 2 who identified as independent were randomly assigned to either the Democrat or Republican target groups in order to minimize bias due to any non-random factors impacting partisan identification.

### Measures

#### Perceived recognition

Participants indicated whether they believed they had seen the headline before on a 7-point scale from “strongly disagree” to “strongly agree.” See Table 2 for descriptive statistics across posts per study.

**Table 2:** Descriptive statistics by platform.

	Study 1: fake content *M (SD)*	Study 2: real content *M (SD)*	Study 2: outlet (General) *M (SD)*
*Perceived recognition*			
Alternative (Just The News)	3.02 (2.16)	3.32 (2.07)	3.61 (1.85)
Consonant (Fox News or CNN)	3.11 (2.17)	3.46 (2.05)	5.11 (1.70)
Dissonant (Fox News or CNN)	3.06 (2.16)	3.41 (2.10)	4.68 (1.86)
Neutral mainstream (ABC News)	*NA*	3.45 (2.07)	4.95 (1.65)
*Perceived accuracy*			
Alternative (Just The News)	3.83 (1.93)	4.31 (1.66)	3.88 (1.55)
Consonant (Fox News or CNN)	4.01 (1.84)	4.44 (1.62)	4.64 (1.63)
Dissonant (Fox News or CNN)	4.02 (1.87)	4.37 (1.62)	3.56 (1.90)
Neutral Mainstream (ABC News)	*NA*	4.43 (1.60)	4.41 (1.64)

CNN and Fox were classified as consonant or dissonant outlets according to the participant’s partisanship.

#### Perceived accuracy

Following Pennycook and Rand (2019), participants rated their agreement with the statement, “To the best of my knowledge, the above headline is accurate,” on the same scale as for perceived recognition (see Table 2 for descriptive statistics).

#### Outlet-level measures

In Study 2, per each outlet (CNN, Fox, Just the News, and ABC), participants completed measures of the extent to which they had seen or heard stories from *that outlet* (outlet-level perceived recognition) and whether stories from that outlet were accurate (outlet-level perceived accuracy).

#### News media literacy

News media literacy (NML) was assessed utilizing the short form of Ashley et al.’s (2013) scale developed by Jones-Jang et al. (2021). Participants rated their agreement with a set of statements on a 5-point scale from “Strongly disagree” to “Strongly agree.” Statements included: “The owner of a media company influences the media content,” and “Individuals can find news sources that reflect their own political values.”

For Study 1, Factor structure was assessed using an Exploratory Factor Analysis with principal axis factoring and direct oblimin rotation. This approach yielded a one-factor model explaining 41.91% of the variance (McDonald’s ω: 0.809; Cronbach’s α: 0.807). On average, participants agreed with scale items, *M* = 3.92 (*SD* = 0.70).

In Study 2, once again, an EFA yielded a one-factor model which explained 46.31% of the variance (McDonald’s ω: 0.837; Cronbach’s α: 0.836.) On average, participants agreed with scale items, *M* = 3.92 (*SD* = 0.73).

#### Covariates

As described in detail in online Supplementary material Appendix C, and detailed in Table 1, several covariates were selected to help isolate the impact of NML. As noted in the Results section, models were also run excluding covariates and focusing on NML, partisan extremity, and the experimental conditions, only.

#### Partisan extremity

Following a simplified version of Weisberg’s (1983) approach to measuring partisanship, participants rated their partisanship on a scale from Strong Democrat to Strong Republican, with middle categories including “Not Very Strong Democrat [Republican], “Lean Democrat [Republican]” and “Independent.” Folded at the midpoint of the scale (independent)” these categories were then utilized to create the measure of partisan extremity, with independent coded as 0, leaners coded as 1, not very strong partisans coded as 2, and strong partisans coded as 3.

### Data analysis

All models were multi-level regression models with impressions of posts nested within the participant. See online Supplementary material Appendix D for Intraclass Correlation Coefficients per dependent variable. See online Supplementary material Appendix E for power analyses.

## Results

A summary of findings per each hypothesis and research question per each study can be found in Table 3.

**Table 3:** Hypotheses and research questions.

H or RQ	Study 1 Fake News	Study 2 Real News
H1a: Alternative (vs. mainstream) outlets are perceived to have general content that is *less recognizable*.		
	NA	Supported
H1b: Alternative (vs. mainstream) outlets are perceived to have general content that is *less accurate*.		
	NA	Supported
H2a: Specific partisan-consonant fake {real} news posts from alternative (vs. mainstream) outlets are perceived to be *less recognizable*.		
	Not supported	Supported
H2b: Specific partisan-consonant fake {real} news posts from alternative (vs. mainstream) outlets are perceived to be *less accurate*.		
	Supported	Supported
H3a: For an alternative news outlet (versus a mainstream news outlet), higher NML users perceive its general content to be *less recognizable*.		
	NA	Supported
H3b: For an alternative news outlet (versus a mainstream news outlet), higher NML users perceive its general content to be *less accurate*.		
	NA	Supported
H4a: When specific partisan-consonant fake {real} news content is associated with an alternative news outlet (versus a mainstream news outlet), higher NML users perceive this content to be *less recognizable*.		
	Supported	Not Supported
H4b: When specific partisan-consonant fake {real} news content is associated with an alternative news outlet (versus a mainstream news outlet), higher NML users perceive this content to be *less accurate*.		
	Supported	Not Supported
RQ2: Are impacts per (a) H3a, (b) H3b, (c) H4a, or (d) H4b further contingent on users’ partisan extremity?		
	H3a: NA H3b: NA H4a: No H4b: Yes	H3a: Yes H3b: No H4a: No H4b: Yes

### Main effects of outlet status

First, models were run excluding covariates, given the experimental design. Main effects per each outcome and their associated t-tests are included in Table 4. For full regression tables including covariates, see online Supplementary material Appendix F. In summary, *general* content from the alternative outlet was perceived to be both less recognizable and less accurate. Parallel results were found for specific real news posts. However, for fake news content, outlet status did not impact perceived recognition but did impact perceived accuracy. See the Discussion for full descriptive interpretation of results.

**Table 4:** Main effects of outlet status (summary of findings).

	Fake content	Real content	Outlet (General)
Outlet status	DV: perceived recognition		
Alternative = 1; Well-known = 0	*b* = −0.07 *t*(2024.00) = −1.14 *p* = 0.253	*b* = −0.12 *t*(4190.00) = −2.73 *p* = 0.006^**^	*b =* −1.30 *t*(4191.00) = −29.18 *p* < 0.001^***^
Outlet Status	DV: perceived accuracy		
Alternative = 1; Mainstream = 0	*b* = −0.19 *t*(2024.00) = −3.35 *p* < 0.001^***^	*b* = −0.10 *t*(4190.00) = −2.80 *p* = 0.005^**^	*b* = −0.32 *t*(4191.00) = −7.02 *p* < 0.001^***^

^*^p < 0.05, ^**^p < 0.01, ^***^p < 0.001.

### Effects contingent on NML

Next, models were constructed including NML and the interaction of NML and outlet status. First, Table 5 notes interactions terms and their significance values.

**Table 5:** Interaction of outlet status and NML.

	Study 1: fake news	Study 2: real news	Study 2: outlets (General)
Outlet status	DV: perceived recognition		
Alternative = 1; Mainstream = 0	*b* = −0.20 *t*(2024) = −2.45 *p = 0*.014^*^	*b* = 0.01 *t* (4191.00) = 0.23 *p* = 0.821	*b* = −0.81 *t*(4191.00) = −13.56 *p* < 0.001^***^
Outlet status	DV: perceived accuracy		
Alternative = 1; Mainstream = 0	*b* = −0.18 *t*(2024) = −2.26 *p* = 0.024^*^	*b* = −0.02 *t*(4191.00) = −0.46 *p* = 0.649	*b* = −0.14 *t*(3191.00) = −2.19 *p* = 0.029^*^

^*^p < 0.05, ^**^p < 0.01, ^***^p < 0.001.

**Figure 1:**
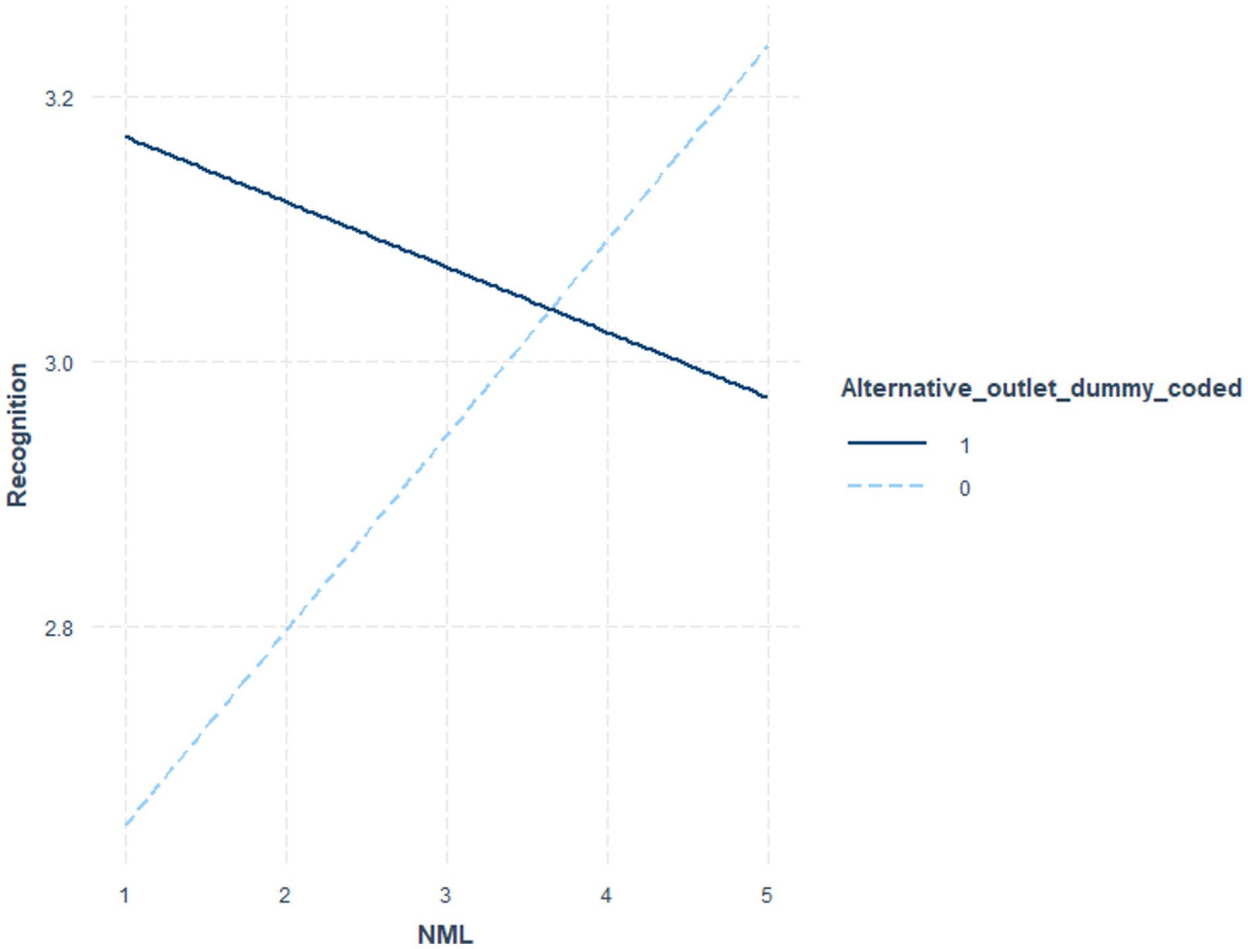
Perceived recognition of fake news posts by NML and outlet status.

**Figure 2:**
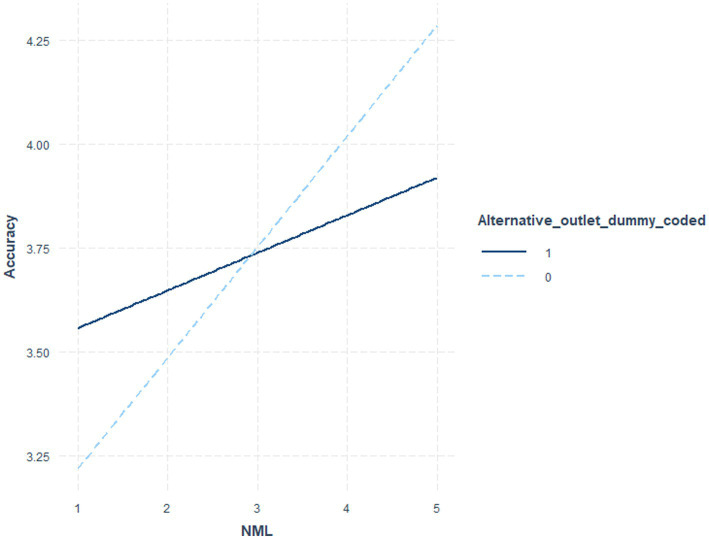
Perceived accuracy of fake news posts by NML and outlet status.

**Figure 3:**
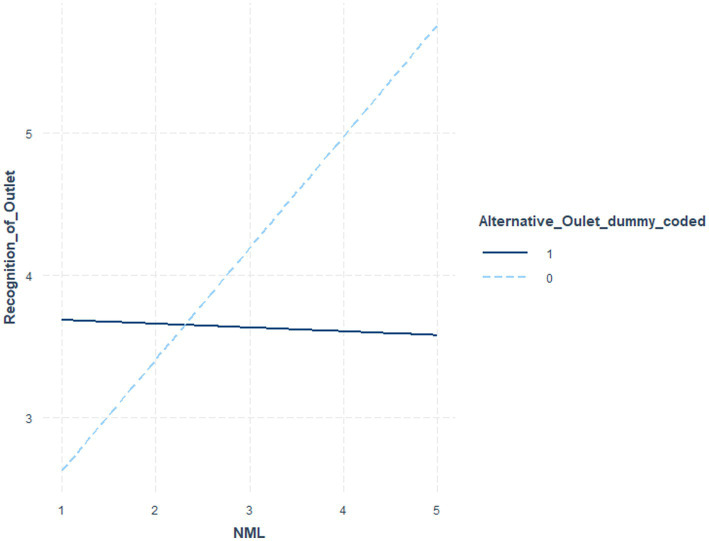
Perceived recognition of outlet by NML and outlet status.

**Figure 4:**
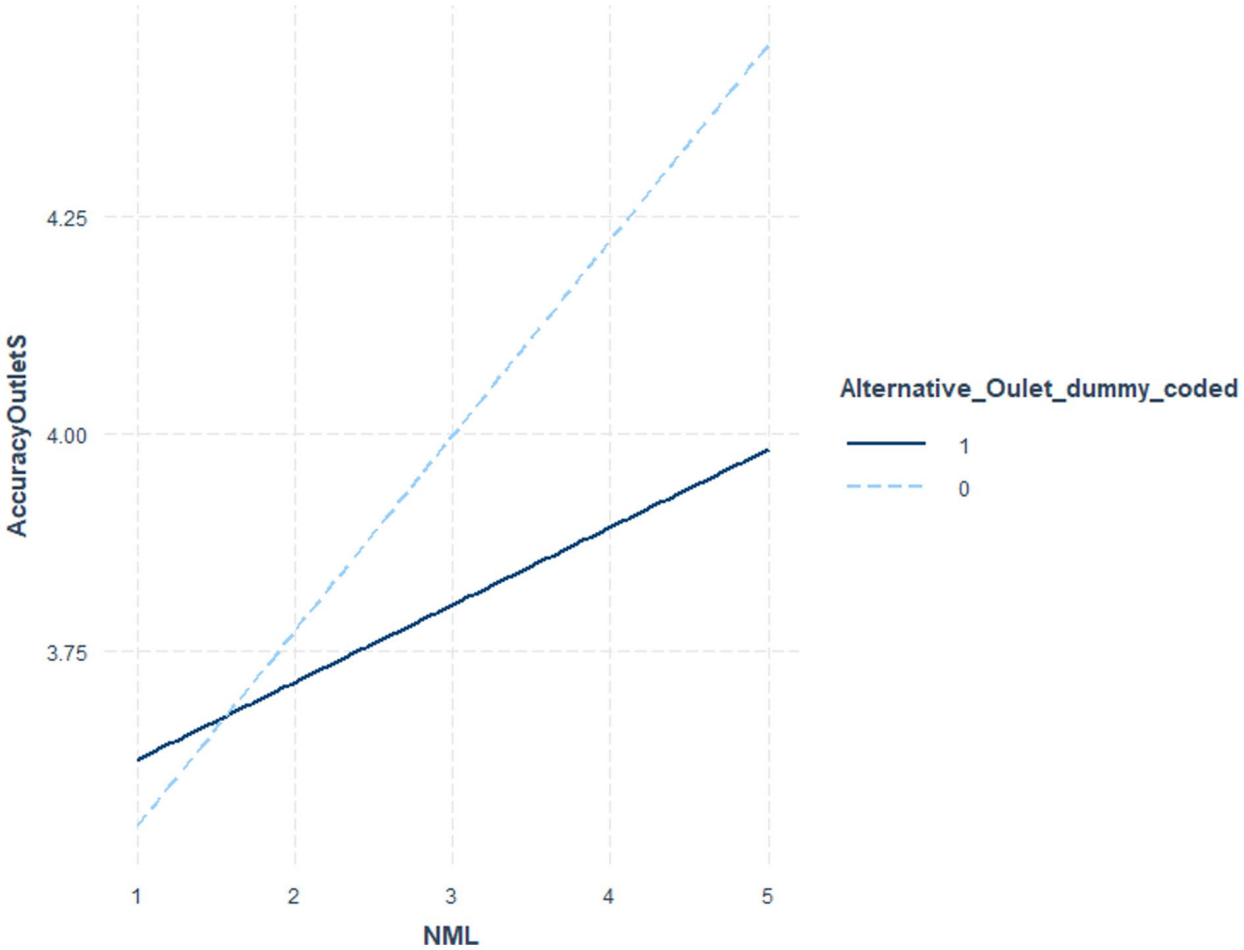
Perceived accuracy of outlet by NML and outlet status.

Table 6 indicates the coefficients and significance values for outlet status at low (*1 sd* below the mean), moderate (mean), and *high* (1 *sd* above the mean) NML, where the interaction term was significant. Table 7 utilized NML as the focal variable. Figure 1 illustrates these post-level contrasts for perceived recognition and Figure 2 illustrates these post-level contrasts for perceived accuracy. Figures 3, 4 illustrate these same contrasts in terms of *outlet-level* perceptions.

**Table 6:** Effects of outlet status as contingent on NML.

		Study 1: fake news	Study 2: real news	Study 2: outlet (General)
IV: outlet status	NML	DV: perceived recognition		
Alternative = 1; Mainstream = 0	Low Moderate High	*b* = 0.07 *b* = −0.07 *b* = −0.21^*^		*b* = −0.71^***^ b = −1.30^***^ b = −1.89^***^
IV: outlet status	NML	DV: perceived accuracy		
Alternative = 1; Mainstream = 0	Low Moderate High	*b =* −0.06 *b =* −0.19^**^ b = −0.31^***^		*b* = −0.22^**^ b = −0.32^***^ b = −0.42^***^

^*^p < 0.05, ^**^p < 0.01, ^***^p < 0.001.

**Table 7:** Effects of NML as contingent on outlet status.

		Study 1: fake news	Study 2: real news	Study 2: outlet (General)
IV: NML	Outlet status	DV: perceived recognition		
NML	Alternative mainstream	*b* = −0.05 *b* = 0.15		*b* = − 0.03 *b* = 0.78^***^
IV: NML	Outlet status	DV: perceived accuracy		
NML	Alternative mainstream	*b* = 0.09 *b* = 0.27^***^		*b* = 0.09 *b =* 0.23^***^

^*^p < 0.05, ^**^p < 0.01, ^***^p < 0.001.

In summary, for *general* perceptions of outlet content and for *specific* impressions of fake news posts, those higher in NML both perceived the content to be less recognizable and less accurate. Parallel results were found for specific fake news posts. Notably, however, as illustrated in Figures 1–4, for perceived accuracy and perceived recognition, the impacts of NML were often on perceptions of the mainstream news outlets rather than the alternative news outlet. See the Discussion for a descriptive interpretation of these results.

### Effects contingent on NML and partisan extremity

**Figure 5:**
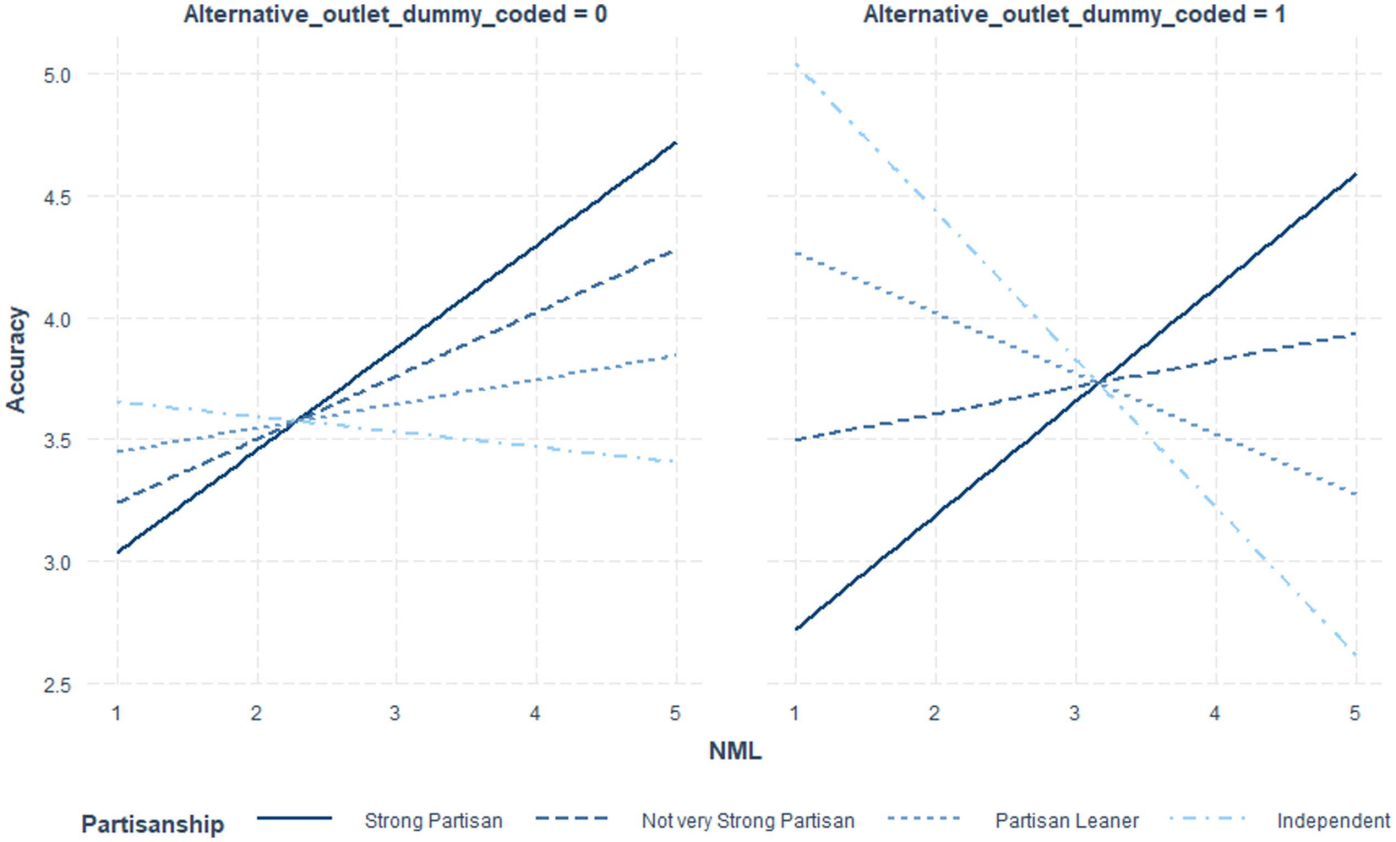
Impact of news media literacy on perceived accuracy of fake news posts by outlet status and political extremity.

**Figure 6:**
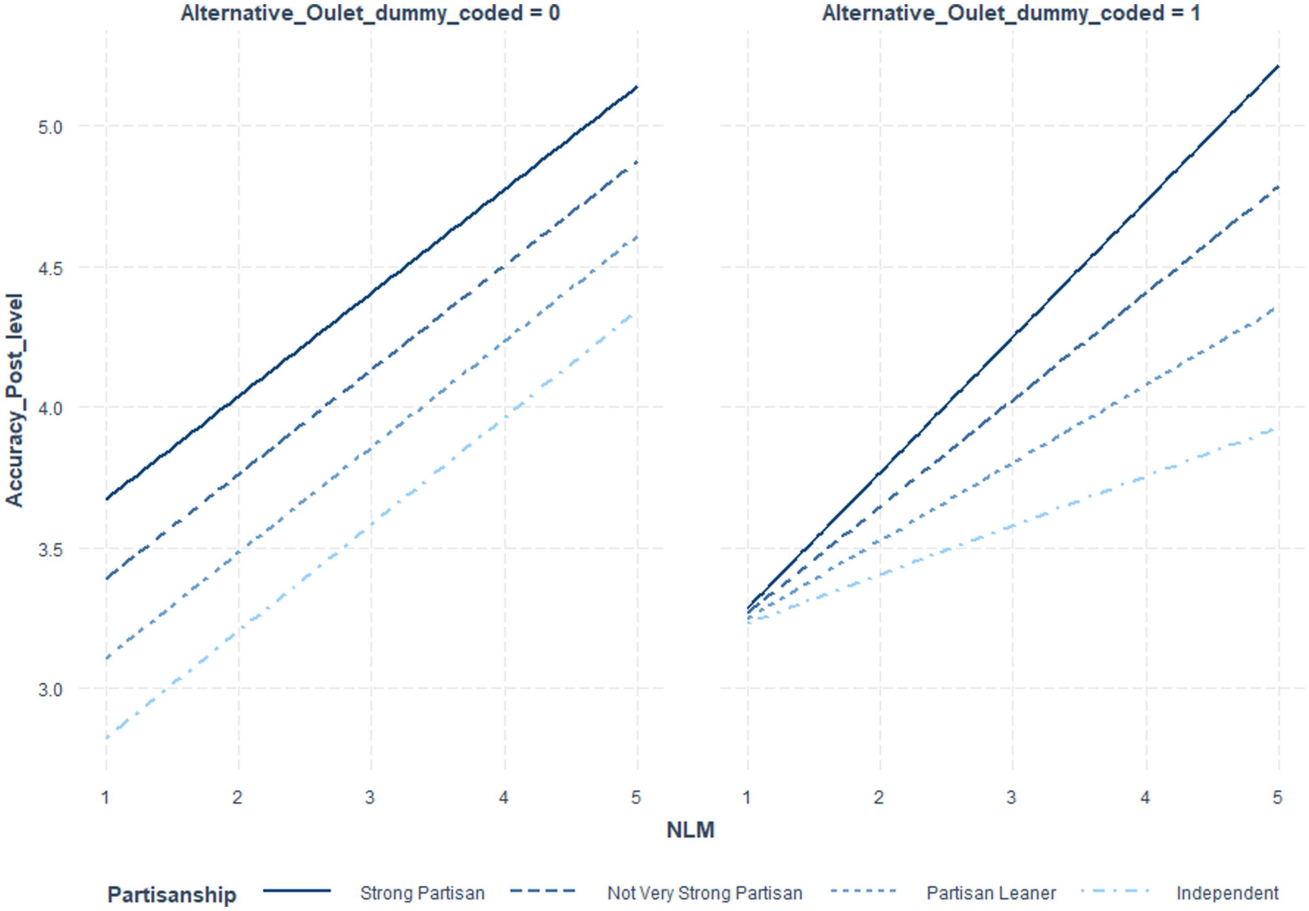
Impact of news media literacy on perceived accuracy of real news posts by outlet status and political extremity.

**Figure 7:**
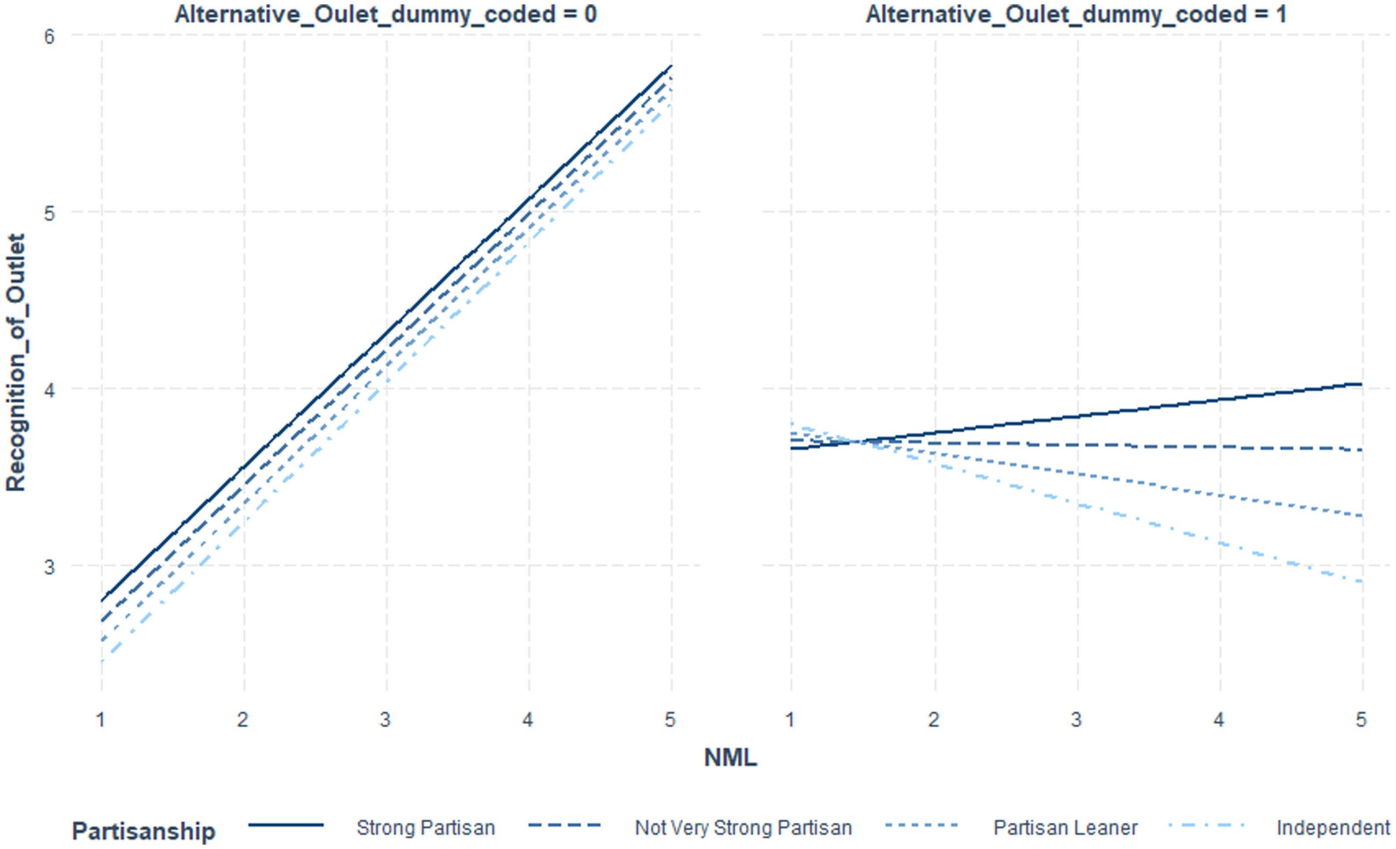
Impact of news media literacy on perceived recognition per outlet by outlet status and political extremity.

Once again, multi-level models were constructed, this time including a three-way interaction between outlet status, NML, and partisan extremity. See Table 8 for these coefficients and their significance values. See Table 9 for values by partisan extremity and NML. See Table 10 for an alternative presentation of the data, treating news media literacy as the focal variable. See Figures 5–7 for illustrations. See Supplementary Appendix G for details per each significance test.

**Table 8:** Interaction terms for outlet status, NML, and partisan extremity.

	Study 1: fake news	Study 2: real news	Study 2: outlet (General)
Outlet status	DV: perceived recognition		
Alternative = 1; Mainstream = 0	*b* = 0.13 *t*(2024.00) = 1.92 *p* = 0.055	*b* = 0.03 *t*(4191.00) = 0.67 *p* = 0.505	*b* = 0.12 *t*(4191.00) = 2.40 *p* = 0.017^*^
IV	DV: perceived accuracy		
Alternative = 1; Mainstream = 0	*b* = 0.20 *t*(2024.00) = 3.01 *p* = 0.003^**^.	*b* = 0.11 *t*(4191.00) = 2.56 *p* = 0.011^*^	*b* = 0.02 *t*(4191.00) = 0.40 *p* = 0.686

^*^p < 0.05, ^**^p < 0.01, ^***^p < 0.001.

**Table 9:** Effects of outlet status contingent on NML and partisan extremity (summary of findings).

		Study 1: fake news	Study 2: real news	Study 2: outlet (general)
		*Independents*		
Outlet status	NML	DV: perceived recognition		
Alternative = 1; Mainstream = 0	Low Moderate High			*b* = −0.88^***^ b = −1.62^***^ b = −2.37^***^
IV	NML	DV: perceived accuracy		
Alternative = 1; Mainstream = 0	Low Moderate High	*b* = 0.15 *b* = −0.24^*^ b = −0.62^***^	*b* = −0.04 *b* = −0.20^**^ b = −0.35^***^	
		*Partisan Leaners*		
IV	NML	DV: perceived recognition		
Alternative = 1; Mainstream = 0	Low Moderate High			*b* = −0.79^***^ b = −1.45^***^ b = −2.11^***^
IV	NML	DV: perceived accuracy		
Alternative = 1; Mainstream = 0	Low Moderate High	*b* = 0.03 *b* = −0.22^**^ b = −0.46^***^	*b* = −0.07 *b* = −0.15^**^ b = −0.22^**^	
		*Not very strong partisans*		
IV	NML	DV: perceived recognition		
Alternative = 1; Mainstream = 0	Low Moderate High			*b* = −0.69^***^ b = −1.27^***^ b = −1.84^***^
IV	NML	DV: perceived accuracy		
Alternative = 1; Mainstream = 0	Low Moderate High	*b* = −0.09 *b* = −0.20^***^ b = −0.30^***^	*b* = −0.11 ^ʈ^ *b* = −0.10^**^ b = −0.09	
		*Strong partisans*		
IV	NML	DV: perceived recognition		
Alternative = 1; Mainstream = 0	Low Moderate High			*b* = −0.60^***^ b = −1.09^***^ b = −1.58^***^
IV	NML	DV: perceived accuracy		
Alternative = 1; Mainstream = 0	Low Moderate High	*b* = −0.21^ʈ^ *b* = −0.18^*^ b = −0.14	*b* = −0.14 *b* = −0.05 *b* = 0.03	

^*^p < 0.05, ^**^p < 0.01, ^***^p < 0.001, ^ʈ^*p* < 0.10.

**Table 10:** Effects of NML contingent on partisan extremity and outlet status (summary of findings).

		Study 1: fake news	Study 2: real news	Study 2: outlet (general)
		DV: perceived recognition		
IV	Partisanship	*Mainstream outlet*		
NML	Strong partisan Not very strong partisan Partisan leaner independent			*b* = 0.76^***^ b = 0.77^***^ b = 0.78^***^ b = 0.79^***^
IV	Partisanship	*Alternative outlet*		
NML	Strong partisan Not very strong partisan Partisan leaner independent			*b* = 0.09 *b* = −0.01 *b* = −0.12 *b* = −0.22 ^*^
		DV: perceived accuracy		
IV	Partisanship	*Mainstream outlet*		
NML	Strong partisan Not very strong partisan Partisan leaner independent	*b* = 0.42^***^ b = 0.26^***^ b = 0.10 *b* = −0.06	*b* = 0.37^***^ b = 0.37^***^ b = 0.38^***^ b = 0.38 ^***^	
IV	Partisanship	*Alternative outlet*		
NML	Strong partisan Not very strong partisan Partisan leaner independent	*b* = 0.47^***^ b = 0.11 *b* = −0.25^*^ b = −0.61^***^	*b* = 0.48^***^ b = 0.38^***^ b = 0.28^***^ b = 0.17^ʈ^	

^*^p < 0.05, ^**^p < 0.01, ^***^p < 0.001, ^ʈ^ = *p* < 0.10.

In summary, the three-way interaction was significant for perceived recognition of outlet content, generally. Additionally, it was significant for the perceived accuracy of specific fake and real news posts. Probing the interactions further, as illustrated in Figures 5–7 and Table 10, for independents only, those higher in NML both perceived the mainstream outlets to be more recognizable and perceived the alternative outlet to be less recognizable. For all others, impacts were driven by the increased perceived recognizability of the mainstream outlets. Turning to perceptions of accuracy of the specific fake and real news posts, while NML was associated with perceiving fake news content attributed to alternative outlets as less accurate, for strong partisans, this relationship was reversed. See the Discussion for a descriptive interpretation of results. For real news content, strong partisans demonstrated a parallel pattern.

### Exploratory analyses

Unlike in real world contexts, participants were not able to actively verify headlines before indicating their likelihood of sharing the associated posts. This limitation makes inferences about sharing likelihood more difficult. For example, while more news media literate participants were capable of forming perceptions of the accuracy of news headlines, they were not then able to verify this accuracy before committing to sharing. As detailed in Appendix H, in both studies, those higher in news media literacy reported significantly lower levels of sharing likelihood, suggesting that they were indeed more cautious. Note, however, that, as predicted by Pennycook and Rand’s (2021) account of fake news sharing, both perceived recognition and perceived accuracy has significant, positive, relationships with sharing likelihood in Study 1 and Study 2.

## Discussion

Two studies, employing *fake news* content and *real news* content respectively, sought to observe the interactive impacts of user psychology and news outlet cues on perceptions of news content posted on social media. One main focus of this analysis was on perceived *recognition.* Recognition was targeted as a key variable, because, from a normative perspective, the current analysis argues that people should indeed be suspicious of content if they do not recognize it or its source.

This psychological state, suspicion, is central to our arguments. Confronted with content from an unfamiliar outlet, a user who is instead neutral but open-minded may be swayed by the plausibility of its content, with plausibility judgments in turn being biased by prior psychological commitments (e.g., partisan bias). A fully suspicious user, in contrast, should doubt content attributed to the outlet even when being told what he wants to hear.

While we acknowledge that this suspicion could lead to doubting even *real news* content and thus lower overall discernment between fake and real news (e.g., Dias et al., 2020), we argue that for the overall benefit of society, it is important that citizens be able to form beliefs that are not just *true* but also well-*justified*. The pathway to justification depends on a variety of factors. However, in the age of fake news, source recognition is a powerful one. Unfamiliar outlets could, potentially, entirely fabricate information. It is thus wise to view their content with greater suspicion, until that content can be independently verified. Indeed, it is reasonable to direct such suspicion even at mainstream outlets. However, for a user who lacks the time or ability to verify every piece of information, there is less risk in trusting content from a mainstream news outlet, as that content is consumed by many and thus more likely to have its errors detected and corrected.

Beyond perceived recognition of the outlet, studies 1 and 2 examined perceived recognition of specific pieces of news content. Following an upswell of research on the role of ease of processing (i.e., fluency) in belief formation and information evaluation (Shulman and Bullock, 2019), we considered it important to investigate perceived recognition. Notably, certainty that one has or has not seen the content before could create a sense of fluency, leading to outright rejection or acceptance. Uncertainty or ambivalence, in contrast, could generate disfluency and promote open-minded skepticism. While the current work cannot address these downstream consequences, it can speak to the interaction of post-level and user-level factors that predict perceived recognition.

In both studies, perceived recognition was considered alongside perceived accuracy. It should be noted, here, that accuracy perceptions may be more strongly subject to partisan bias, beyond any relationship with perceived recognition of source or content. As discussed below, while post-level and user-level factors often impacted perceived accuracy and perceived recognition similarly, unique patterns did emerge that provided insights, in particular, into the role of partisan bias in shaping our responses to social media news content.

Last, whether examining perceived recognition or perceived accuracy, News Media Literacy played a fundamental role in shaping participant’s perceptions.

### Perceived recognition

First, participants did indeed feel unfamiliar with content from Just The News, relative to more common news outlets. This served, in part, as a manipulation check. H1a was supported, as was H3a. Not only did participants report lower levels of perceived recognition regarding Just The News on average, those higher in News Media Literacy demonstrated a more pronounced trend. This trend was driven by greater perceived recognition of mainstream news outlets.

Interestingly, per RQ1, this pattern was further moderated by political extremity. Specifically, regarding perceptions of the alternative news outlet, for independents, News Media Literacy was associated with reporting *even* lower levels of recognition.

In other words, while participants generally reported lower levels of recognition of the alternative news outlet, and there was no direct evidence for a motivated reasoning process, independents, may have been even more sharply aware that this was, to them, an unfamiliar source of information. Such a clear lack of familiarity with the source could in turn lead them to more easily dismiss its content.

This finding regarding general impressions of the outlet helps to contextualize the results per the specific fake and real news posts used as stimuli in Study 1 and Study 2 respectively, as tested per H2a and H4a.

Notably, in Study 2, attributing real news content to the alternative news outlet vs. the mainstream news outlet was associated with *lower* overall levels of recognition, an impact that was not moderated further. In other words, the outlet cue had a powerful impact on the subjective perception of whether content was recognizable. This finding further supports the notion that feelings of recognition are in part guesses based on a variety of factors and sometimes at odds with real experiences. This finding also supports theorizing regarding the bidirectional relationship between perceptions of source and of content.

Interestingly, in models employing covariates to test the robustness of the effects, higher amounts of self-reported news exposure (averaged across channels) the prior week was positively associated with perceived recognition of *both* fake and real news posts (see Supplementary Appendix F). Perhaps those who consume a larger amount of news, rather than growing more discerning, can instead become more confused, recalling related content and reasoning that they may in fact have seen even fake news content before.

Alternatively, some participants may have actually been exposed to the content—fake or real. However, this is less likely for the fake news content utilized in Study 1, as this content was sampled from a wide variety of fake news sources, limiting the likelihood that any particular participant would have encountered any particular piece of fake news. The fact that news exposure played a positive predictive role in both studies supports the notion that perceived recognition is more a reflection of the subjective plausibility of having encountered content before than any specific memory of that content.

Study 1’s results per fake news posts help to elucidate this pattern. Here, those higher in News Media Literacy experienced greater recognition of fake news content when it was attributed to mainstream news outlets. In other words, across the current studies, it is likely that participants were neutral as to whether they recognized content attributed to the alternative news outlet but assumed that they might have seen the content before when it was attributed to the mainstream news outlet. More research is needed, however, to disentangle the impacts of specific attributes of the fake vs. real news content, which could further impact perceptions of familiarity.

In summary, mainstream news outlets conferred greater feelings of recognition to their associated content. For fake news content, News Media Literacy magnified this advantage. However, turning to reactions to the outlet, more generally, additional evidence suggests that, for the independents only, News Media Literacy rendered them more sharply skeptical regarding their familiarity with the alternative news outlet, and thus may offer independents better protection against embracing erroneous content.

#### Conclusions regarding perceived recognition and practical implications

Beyond highlighting the value of News Media Literacy, these results illustrate the complex and multi-faceted nature of perceived recognition. Future research should seek to disentangle the impacts of no, low, and high subjective feelings of recognition on how content is processed. As discussed per Shulman and Bullock’s (2019) call to integrate more discussion of metacognition into communication theory, it is possible that perceived recognition could have more than a simple, direct, impact on perceived accuracy. It could also impact the amount of processing that a user devotes to a social media news post. A user who is certain that they do not recognize a headline may dismiss it more readily. People who think they recognize it but are not sure may gather more information before making up their minds. A person who is certain that they recognize it may accept it without further effort.

Further, it would be useful to examine factors that impact perceived recognition outside of the more ideal conditions of these experiments, where many of the distractions of social media are removed and users are prompted to give careful consideration to every post. While these ideal conditions were utilized to offer a strong test of concerns about bias, more research is needed as to how these processes would unfold on a more cluttered, every day, social media feed. Optimistically, however, it is possible that the proposed disfluency experienced by those who are not sure whether they recognize outlets or content would motivate seeking out further information despite the observed (e.g., Pearson, 2021) obstacles.

### Perceived accuracy

Perceived accuracy was considered in parallel to perceived recognition. While perceived recognition would be predicted to impact perceived accuracy, other factors, such as the plausibility or desirability of content, as well as general perceptions of its associated news outlet, could shape accuracy judgments.

Considering general impressions of each outlet per H1b, perceived accuracy regarding general content was indeed lower for the alternative news outlet, an effect that was further amplified by News Media Literacy, per H3b. Specifically, as News Media Literacy increased, so did the perceived credibility of more mainstream news outlets.

Examining *specific* fake and real news content, however, the results grow more complex. First, H2b was supported. Both fake and real news content attributed to the alternative news outlet was perceived to be less accurate. This is, as argued in the introduction, a normatively desirable result, even if it leads to doubting real news.

Further, per H4b, News Media Literacy amplified this pattern, with regards to fake news content only (Study 1). The higher their News Media Literacy, the more participants perceived fake news content to be accurate when it was attributed to mainstream news sources. In other words, News Media Literacy rendered participants more vulnerable to believing in fake news delivered by mainstream news outlets and did not pose a particular protective influence against believing in fake news content from alternative news outlets. That being said, most participants demonstrated less belief in the latter, suggesting the potential for floor effects to have played a role in this analysis.

Considering the role of partisan extremity per RQ1, which had a limited impact on perceived recognition, notable findings emerged for perceived accuracy. Specifically, while partisan extremity did not further impact general perceptions of each outlet, it did shape the perceived accuracy of content attributed to that outlet, in concert with NML.

Considering partisan-favoring fake news content per Study 1, those higher in News Media Literacy perceived this content to be more accurate whether it was attributed to either a mainstream or an alternative news outlet, if they also had strong partisan commitments. Only for independents and those who merely leaned toward a political party did News Media Literacy have a truly protective effect. Turning to the real news content utilized in Study 2, parallel results were found. This suggests that while the judgments of those high in News Media Literacy were indeed impacted by the outlet cue, only those with weaker partisan commitments were truly suspicious. Those with stronger partisan commitments appeared to be neutral but open-minded in a fashion that allowed them to justify embracing partisan-consistent content and even praising an unfamiliar outlet for delivering such content. This pattern is particularly pernicious, as it implies that even for high News Media Literacy partisans, alternative outlets can promote positive brand impressions simply by telling them what they want to hear.

#### Conclusions regarding perceived accuracy and practical implications

News Media Literacy did not protect those with stronger partisan commitments against believing in fake news content, even under so-called ideal conditions in which they were prompted to consider the accuracy of each social media post. However, the picture is still hopeful. News Media Literacy, measured as a set of attitudes, did, on average, predict differential responses to content attributed to the alternative vs. more mainstream news outlets. News Media Literacy training that emphasizes the importance of understanding outlets and their potential biases could thus, potentially, benefit most users.

However, for those partisans who are high in News Media Literacy but defend the accuracy of desirable content, anyway, further intervention may be warranted. News Media Literacy training could be paired with information literacy training and thus emphasize justification: Can individuals point to specific types of evidence in favor of the headline? This information literacy would however need to be tailored to different types of news content, as more general information literacy may not prepare the user for evaluating specific content.

To further encourage this more effortful process, training could be combined with a motivation-focused intervention targeting strong partisans. Such an intervention might specifically offer arguments regarding both the intragroup consequences of sharing fake news (e.g., sowing doubt and confusion within one’s own ranks) and its intergroup consequences (providing ammunition for criticism by rival partisans). These arguments, in combination with news media literacy and specific information literacy training, could be successful in tackling the pervasive problem of partisan misinformation generally and fake news specifically.

However, any interventions would have to contend with the distracting conditions of the real-world social media environment. While intervention-related content could be introduced into real world social media feeds, it is likely that additional effort is warranted. For example, where a platform gives its users power to shape interactivity affordances (e.g., as “polls”) or reward positive contributions (e.g., badges), this power could be harnessed to encourage and reward users when they offer high quality justifications for sharing content.

### Limitations

These findings may be specific to the US context, which has the most politically polarized news audiences across 12 countries (Fletcher et al., 2020) and has demonstrated greater susceptibility to online disinformation (Humprecht, 2023). For example, in US samples, political beliefs can be strongly tied to social reward and punishment (see Sude and Knobloch-Westerwick, 2022, for a review), which may explain why even unfamiliar partisan-aligned content is embraced as true.

These studies, of course, were not without other limitations. One major limitation is that they cannot directly address the proposed psychological processes that motivate attending to and interpreting the outlet cue. A more detailed examination of these processes is warranted. A second major limitation is that fake and real news content employed in each study may have differed from one another on attributes such as topic and style. Last, NML is measured, not manipulated. Future studies could employ NML training. A last limitation is that participants could not click through to view the articles themselves, which may have disrupted the verification process of high NML individuals.

## Overall conclusion

Two studies with large, diverse, samples of adult participants from the US examined post-level and user-level factors that could impact perceptions of *fake* and *real* news content encountered online. Building upon Pennycook and Rand’s (2021) contrast between their confusion-based and inattention-based accounts of fake news sharing, the current work probed factors that could both ameliorate and intensify confusion.

The first factor examined was whether a news headline was associated with an alternative vs. mainstream news outlet. Notably, participants were sensitive to the news outlet cue, and indeed were less confident that they recognized content if it was attributed to an unfamiliar, alternative, news outlet. They also were generally suspicious of the accuracy of this content, relative to content from mainstream sources.

Turning to user-level factors, News Media Literacy (Ashley et al., 2013) was identified as promoting sensitivity to the outlet cue and did, on average, have a normatively desirable impact on both perceived recognition and perceived accuracy.

However, a second, user-level factor, partisan extremity, was identified as having a countervailing effect. The pattern of results was congruent with the following claim: One cannot rely on partisans to self-assess their justifications for deciding whether partisan-consistent content is accurate or not. Even when they are motivated to do so, they may ironically become even more strongly committed to perceiving content as accurate, a pattern observed more broadly in social psychological research on human bias (Chien et al., 2014).

In light of these findings, a multi-part intervention strategy targeting those with partisan commitments was proposed.

While further research is needed, it is possible to imagine a world where fake news outlets, which can produce outright falsehoods, are ignored and, instead, the most evidence-rich content by real news outlets (alternative or mainstream) instead spreads throughout the information ecosystem. While this may seem like a fantasy, careful attention to user psychology as well as the vagaries of the social media information environment, combined with investing in news media literacy and topic-tailored information literacy training, could help get us there.

## Data availability statement

The raw data supporting the conclusions of this article will be made available by the authors, without undue reservation.

## Ethics statement

The studies involving humans were approved by the Institutional Review Board at Tel Aviv University. Contact Einat Berlowitz – Coordinator. Tel: 03-6409673. Email: ethicsbe@tauex.tau.ac.il. The studies were conducted in accordance with the local legislation and institutional requirements. The participants provided their written informed consent to participate in this study.

## Author contributions

DS: experimental design, analyses, and manuscript writing. GS and SD-G: experimental design and manuscript writing. All authors contributed to the article and approved the submitted version.

## Conflict of interest

The authors declare that the research was conducted in the absence of any commercial or financial relationships that could be construed as a potential conflict of interest.

The handling editor GN declared a past co-authorship with the author SD-G.

## Publisher’s note

All claims expressed in this article are solely those of the authors and do not necessarily represent those of their affiliated organizations, or those of the publisher, the editors and the reviewers. Any product that may be evaluated in this article, or claim that may be made by its manufacturer, is not guaranteed or endorsed by the publisher.
