# Supplementary material for: True, justified, belief? Partisanship weakens the positive effect of news media literacy on fake news detection
Source: Front Psychol. 2023 Sep 26;14:1242865. doi: 10.3389/fpsyg.2023.1242865 (PMC10562704; doi:10.3389/fpsyg.2023.1242865)
Supplement: Supplementary file 1 [file Data_Sheet_1.docx]

**Appendix A**

**Study 1 Stimuli Pretest**

**Overview**

Headlines and graphics from 22 real world political and satire articles containing misinformation were selected for the pretest: seven consonant for Republicans, seven consonant for Democrats, three unrelated to political or other controversial topics, and five on controversial health topics. These headlines and graphics were presented as Facebook posts – displaying media brand, headline, and image – with a blacked-out profile picture, name, and date. Each participant saw a randomly selected set of six posts: two consonant for Republicans, two consonant for Democrats, one on a neutral topic, and a last about a controversial health topic.

**Participants**

239 Amazon Mechanical Turk workers completed the study and identified as Republican or Democrat or, if independent, leaning Republican or Democrat. 7.5% of participants reported being between the ages of 18 and 24, 37.2% between 25 and 34, 20.1% between 35 and 44, 26.8% between 45 and 54, 6.3% between 55 and 64, and 2.1% reported being 65 or older. 59.8% identified as male, 39.7% as female, and 0.4% as "other." 62.3% of participants identified as Democrats and 37.7% as Republicans.

**Measures**

Following seeing each post, participants reported whether they had seen or heard about the story before. Participants then rated each article on its accuracy, fairness, trustworthiness, and persuasiveness. Finally, participants classified each post as favorable to Democrats or Republicans.

**Results**

For the main study, posts were selected that participants agreed favored the respective political parties, and that were equivalent in accuracy, fairness, trustworthiness, and persuasiveness. To evaluate stimuli, Republican-identifying and Democrat-identifying participants were considered separately. Stimuli were selected that were (a) not significantly different in terms of accuracy, fairness, trustworthiness, and persuasiveness and (b) perceived by both Republican and Democrat participants, considered separately, as clearly favoring one party over the other, (*p* = .016 and *p* < .001 respectively.)

**Study 2 Stimuli Pretest**

**Pretest 1**

***Overview***

Amazon’s Mechanical Turk Workers viewed four mock Facebook posts of real news stories, constructed using real headlines and leads from a variety of sources (see Appendix C). Headlines were selected based on whether they were favorable towards the Democratic party or favorable towards the Republican party. They covered various topics, from immigration to the January 6^th^ insurrection. However, because researcher judgments about favorability could be biased, each participants viewed four stimuli, two that the researcher judged to favor Democrats, two that researcher judged to favor Republicans. Each of the respective posts was randomly drawn from one of two pools including eight posts deemed to favor the relevant party. All posts were constructed such that dates and source information were blacked out. Participants rated each post on several distractor dimensions and one target dimensions: the political party that the post most appeared to favor.

***Participants***

131 participants were recruited and reported either identifying as Democrat or Republican or, if independent, leaning Democrat or Republican. However, to ensure data quality, responses were checked for straight-lining, using the simple non-differentiation method (Kim et al., 2019). 18 participants were excluded because they, when rating posts, selected the same response option for every measure, across at least two of the four posts. Of the remaining 113 participants, 84.1% identified as Democrats or, if independent, leaning Democrat. Note, because of this imbalance, a second pretest, including only Republican participants and stimuli deemed to be Republican favoring, was then conducted. Participants ranged in age from between 18-24 years old to between 65-74 years old. The median age range selected was between 25 and 34 years old. Additionally, 71.7% of the sample identified as male.

***Measures***

The target measure per stimulus was “In your opinion, do you think the post is” with participants responding on a 5-point scale from “In favor of Democrats” to “In favor of Republicans.” Participants were also asked, “Have you seen or heard this story before?,” as well as asked, “To the best of your knowledge, is the post” followed by adjectives: accurate, fair, trustworthy, and persuasive.

***Results***

Stimuli were selected based on the results of a one-sample t-test, with the goal of retaining posts that Democrat or Democrat-leaning participants perceived to clearly favor the Democrats and that Republican-leaning participants perceived to clearly favor the Republicans. One-sample t-tests were conducted per stimulus, examining whether stimuli were significantly different from the neutral midpoint of the 5-point scale. Notably, Democrats perceived 5 stimuli as significantly favorable towards Democrats. Of these, one had been identified by the researchers as likely favoring the Republicans, as it featured the Democrats blocking a Republican bill concerning COVID-19 testing. This more multi-facetted stimulus was not retained for use in the main study.

Turning to Republican participants, the relatively low number of participants necessitated a second pre-test devoted to only Republican participants. However, to increase the likelihood of a successful second pre-test, relatively successful stimuli from the first stimuli were retained for the second pretest. Three stimuli had mean ratings that were above the scale’s midpoint (*M* = 4.25; *M*  = 3.75; M = 3.50), and therefore, on average, favorable to Republicans, although not significantly so. Note, responses from this first pretest were combined with responses from the second pretest to facilitate stimuli selection.

**Pretest 2**

***Overview***

Only Republican Amazon’s Mechanical Turk workers were included in this additional pretest. Three stimuli that most favored Republicans from the first round of pretesting were retained, and five novel stimuli were added. The rest of the procedure was identical to the first pretest, with the following exception: Participants only viewed posts that were deemed by researchers to be Republican-favoring.

***Participants***

In addition to the Republican participants collected in the prior pretest, 28 new Republican participants were included, after excluding 12 participants for straight-lining using the simple non discrimination criterion described above. Of these participants, reported ages ranged from 25-34 to 55-64, with the median selected range being 35-44. 67.9% identified as male. In summary, the total number of Republican or Republican leaning participants increased to 46, each viewing between two and four Republican-favoring stimuli for the first and second round of pretesting respectively.

***Measures***

Measures were identical to the first pretest.

***Results***

One-sample t-tests were conducted per stimulus, examining whether stimuli were significantly different from the neutral midpoint of the 5-point scale. Three stimuli were identified as significantly favoring Republicans. An additional stimulus was close to significantly different using a one-tailed test, *p* = .104. A follow up analysis contrasted these stimuli as a group to other Republican-favoring stimuli and yielded a significant contrast of the retained Republican-favoring stimuli (*M* = 3.97, *SD* = 1.19) versus the rejected stimuli (*M* = 3.18, *SD* = 1.11), *t*(126) = -3.60, *p* < .001.

**Appendix B**

**Sources of Fake News Headlines**

**Against Dem. and Pro-Rep**

U.S. House Speaker Nancy Pelosi diverted $2.4 billion from the Social Security fund to cover impeachment costs.

Article:

<https://potatriotsunite.com/impeachbill/?fbclid=IwAR062FVZqgoUKv4qfdosGsZRDnCDT_EGyS6yxlCtiBolzQpeYvzuxF9fLMA>

Fact Checking:

<https://avaazimages.avaaz.org/US_2020_report_1105_v04.pdf>

<https://www.businessinsider.com/most-viewed-fake-news-stories-shared-on-facebook-2019-2019-11#2-nancy-pelosi-diverting-social-security-money-for-the-impeachment-inquiry-9>

<https://www.snopes.com/fact-check/pelosi-impeachment-social-security/>

<https://www.factcheck.org/2019/10/pelosi-didnt-raid-social-security-for-impeachment-inquiry/>

Democrats Vote To Enhance Med Care for Illegals Now, Vote Down Vets Waiting 10 Years for Same Service

Article:

<https://www.westernjournal.com/dems-vote-enhance-med-care-illegals-now-vote-vets-waiting-10-years-service/?utm_source=facebook&utm_medium=conservativetribune&utm_content=2019-10-18&utm_campaign=evergreen&fbclid=IwAR2GAu3KADU-HBeHI4vTCx1G5FhA1yBw5eJkkQIYM3iBAzOqWRzk2g9AU1w>

Fact Checking:

<https://www.businessinsider.com/most-viewed-fake-news-stories-shared-on-facebook-2019-2019-11#7-democrats-vote-to-enhance-med-care-for-illegals-now-vote-down-vets-waiting-10-years-for-same-service-4>

<https://www.snopes.com/fact-check/dems-immigrants-vets-health/>

<https://www.factcheck.org/2019/12/a-misleading-take-on-immigrant-veterans-health-care/>

Trump wins CNN lawsuit

https://leadstories.com/hoax-alert/2020/05/fact-check-trump-wins-500-million-libel-lawsuit-against-cnn.html

**Against REP. and Pro-DEM.**

Trump Say Republicans Are the “Dumbest Group of Voters”

Article:

<http://archive.is/Ap6Hb>

<https://other98.com/>

Fact Checking:

<https://www.snopes.com/fact-check/1998-trump-people-quote/>

<https://leadstories.com/hoax-alert/2019/07/fake-news-donald-trump-not-say-republicans-are-dumbest-group-of-voters.html>

<https://www.factcheck.org/2015/11/bogus-meme-targets-trump/>

Oldies will have to die tweets trump

Article:

<https://www.patheos.com/blogs/laughingindisbelief/2020/05/oldies-will-have-to-die-tweets-trump/>

Fact check:

https://leadstories.com/hoax-alert/2020/05/fact-check-oldies-will-have-to-die-tweets-trump.html

**Real News Headlines**

**Perceived to be Democrat-Favoring**

***ABC News***

*Capitol Police officer suspended after anti-Semitic document spotted near security checkpoint*

https://abcnews.go.com/Politics/capitol-police-officer-suspended-anti-semitic-document-spotted/story?id=76488990

***CNN***

*Biden says Putin 'will pay a price' for Russian efforts to undermine the 2020 US election*

https://www.cnn.com/2021/03/17/politics/joe-biden-vladimir-putin-price-to-pay/index.html

*New US intel report shows Russia, Trump and GOP acolytes have same goas*

https://www.cnn.com/2021/03/17/politics/trump-russia-elections/index.html

*Trump State Department appointee violated his oath of office at Capitol riot, DOJ says*

https://www.cnn.com/2021/03/09/politics/federico-klein-trump-state-department-capitol-riot/index.html

**Perceived to be Republican-Favoring**

***CNN***

*A year into the pandemic, Florida is booming and Republican Gov. DeSantis is taking credit*

https://www.cnn.com/2021/03/17/politics/ron-desantis-covid-florida/index.html

***Fox News***

*Texas lawmakers pass bill requiring national anthem to be played by pro sports teams*

https://www.foxnews.com/politics/texas-lawmakers-bill-national-anthem-played-pro-sports-teams

*Texas mayor sounds alarm about robberies, car chases as migrant surge worsens*

https://www.foxnews.com/politics/texas-mayor-robberies-car-chases-border-migrant-surge-worsens

*Arizona sheriff says Biden border crisis worse than Obama years: 'Like the Wild West'*

https://www.foxnews.com/politics/border-crisis-worse-obama-arizona-sheriff-biden-immigration

**Appendix C**

**Covariates**

**Information Literacy**

Information literacy was assessed by asking participants a series of five knowledge-based questions (Podgornik et al., 2016). For example, participants were asked “Which of the data listed below are ‘raw’ unprocessed data?” and given the following options: share prices published at the end of the trading day, weather maps, population growth data presented in tables, and population growth data presented diagrammatically (in graphs).

**Political Interest**

To assess political interest, participants were asked to rate their agreement with several statements on a 7-point scale from strongly disagree to strongly agree. The statements were: “politics is important to me personally,” “I am sure about my political stance,” “I have vast knowledge about politics,” “I don’t tend to change my political stance,” and “I am interested in politics.”

**Trust in News Media**

Following Strömbäck et al. (2020), trust in the news media was assessed with 5-items, which asked participants to respond on a 7-point scale from strongly disagree to strongly agree. Items included, “The news media is fair when covering the news” and “The news media separates facts from opinions when covering the news.”

**Trust in Institutions**

Trust in Institutions was asking participants to indicate their trust in various institutions on a 7-point scale from “Not at all” to “Completely.” Institutions were as follows: politicians, news media, experts and scientists, supreme court judges, the content other users post on Facebook, the content journalists post on Twitter, right wing news media outlets, and left wing news media outlets.

**Faith in Intuition for Facts**

From Garrett & Weeks (2017), faith in intuition for facts was assessed by asking participants to respond to several statements on a 7-point scale from “strongly agree” to “strongly disagree.” Examples items include, “I trust my initial feelings about the facts,” and “I can usually feel when a claim is true or false even if I can’t explain how I know.”

**Need for Evidence**

From Garrett & Weeks (2017), need for evidence was assessed by asking participants to respond to several statements on a 7-point scale from “strongly agree” to “strongly disagree.” Examples items include, “Evidence is more important than whether something feels true,” and “I need to be able to justify my belies with evidence.”

**Truth is Political**

From Garrett & Weeks (2017), perception that the Truth is Political was assessed by asking participants to respond to several statements on a 7-point scale from “strongly agree” to “strongly disagree.” Examples items include, “Facts are dictated by those in power,” and “Scientific conclusions are shaped by politics.”

**Conspiracy Beliefs**

Conspiracy beliefs were assessed by asking participants to evaluate the veracity of eleven conspiracy theories on a 5-point scale from “Definitely false” to “Definitely true.” Theories includes, “G5 technology poses health risks that are concealed from the public,” “Certain significant events have been the result of the activity of a small group who secretly manipulate world events,” and “The moon landing was faked by NASA.”

**News Exposure**

News exposure was assessed by asking participants to indicate the number of days of exposure they had to news media outlets for each of 10 different categories of channels, including “National network news on television,” “websites/apps of mainstream news organizations,” “podcasts,” and “social media.”

**Appendix D**

**Intraclass Correlation Coefficients**

|  | Study 1 | Study 2 |
| --- | --- | --- |
| Recognition | .53 | .50 |
| Accuracy | .41 | .45 |
| Sharing | .67 | .70 |

**Appendix E**

Power analyses performed with the R-package pwr (Champely, 2018) showed that a 1000 participant study, with 4 observations per participant (4000 observations), and 10 parameters per model, has 90 % power to detect significance in models with *F*^2^ > .005, a very small effect according to Cohen (1988). That calculation, however, assumes a design effect of 1.

The design effect, in this study, estimates the impact of employing a multi-level model versus an OLS regression. It is an indicator of “how the particular design chosen – in our case, the multilevel design – affects the standard error of the parameters” (Snijders, 2005, p. 1573). Design effects greater than 1 represent cases where the multi-level model is less efficient (has larger squared standard errors) than the OLS model. Design effects less than 1 represent cases where the multi-level model is more efficient (has smaller squared standard errors).

To estimate the sample size required for the multi-level model, the design effect is multiplied by the number of required observations dictated by a power analysis that assumes an OLS model. In other words, to detect the same effect, a multi-level model with a design effect of 1.25 would need 5000 observations to an OLS model’s 4000. In cases where the multi-level model is more efficient, with a design effect, for example, of 0.85, only 3400 observations would be needed.

Note that in multi-level models where clusters vary in their number of observations, this analysis grows more complex. The design of the current study allows for the simpler power analysis described above. The design effects for the most complex designs, the 3-way interaction terms, across these studies range between 0.52 and 0.64. Thus, each analysis has 90% power to detect even small effects.

References

Champely, S. (2018). *pwr: Basic functions for power analysis.* R package version 1.2-2. <https://CRAN.R-project.org/package=pwr>

Cohen, J. (1988). *Statistical power analysis for the behavioral sciences.* Hillsdale, NJ: L. Earlbaum Associates.

Snijders, T. A. B. (2005). Power and sample size in multilevel linear models. In: B.S. Everitt and D.C. Howell (Eds.), *Encyclopedia of statistics in behavioral science, Volume 3* (1570–1573). Chicester, England: Wiley.

**Appendix F**

**Study 1 and Study 2 Regression Tables with Covariates**

| Table 1: Perceived Recognition of Fake News Posts | | | | | | |
| --- | --- | --- | --- | --- | --- | --- |
|  | Coef | S.E. | t val. | d.f. | p | VIF |
| (Intercept) | -1.11 | 0.39 | -2.84 | 1027.50 | .005^**^ |  |
| Alternative Ouitlet (dummy-coded) | -0.04 | 0.07 | -0.65 | 2016.00 | .518 | 1.33 |
| NML | -0.02 | 0.08 | -0.33 | 1008.00 | .741 | 1.53 |
| Information Literacy | -0.10 | 0.04 | -2.41 | 1008.00 | .016^*^ | 1.16 |
| Consonant Source  (dummy-coded) | 0.04 | 0.07 | 0.66 | 2016.00 | .508 | 1.33 |
| Democrat-Favoring Stimuli  (dummy-coded) | 0.50 | 0.09 | 5.38 | 1008.00 | <.001^***^ | 1.13 |
| Partisan Extremity | 0.14 | 0.04 | 3.27 | 1008.00 | .001^**^ | 1.18 |
| Political Interest | 0.09 | 0.04 | 2.33 | 1008.00 | .020^*^ | 1.65 |
| Male (dummy-coded) | -0.12 | 0.09 | -1.34 | 1008.00 | .180 | 1.15 |
| Age | -0.08 | 0.03 | -2.57 | 1008.00 | .010^*^ | 1.23 |
| Education | 0.10 | 0.04 | 2.40 | 1008.00 | .017^*^ | 1.26 |
| Trust in Media | 0.15 | 0.03 | 4.30 | 1008.00 | <.001^***^ | 1.84 |
| Trust in Institutions | 0.09 | 0.04 | 2.00 | 1008.00 | .046^*^ | 1.88 |
| Faith in Intuition for Facts | -0.01 | 0.05 | -0.19 | 1008.00 | .852 | 1.40 |
| Need for Evidence | 0.06 | 0.05 | 1.21 | 1008.00 | .225 | 1.53 |
| Truth is Political | 0.09 | 0.04 | 2.43 | 1008.00 | .015^*^ | 1.46 |
| Conspiracy Beliefs | 0.45 | 0.06 | 6.99 | 1008.00 | <.001^***^ | 1.66 |
| News Exposure | 0.22 | 0.03 | 6.43 | 1008.00 | <.001^***^ | 1.83 |

| Table 2: Perceived Accuracy of Fake News Posts | | | | | | |
| --- | --- | --- | --- | --- | --- | --- |
|  | Est. | S.E. | t val. | d.f. | p | VIF |
| (Intercept) | -0.03 | 0.35 | -0.09 | 1031.34 | .931 |  |
| Alternative Outlet  (dummy-coded) | -0.19 | 0.06 | -2.98 | 2016.00 | .003^**^ | 1.33 |
| NML | 0.11 | 0.07 | 1.65 | 1008.00 | .099 | 1.53 |
| Information Literacy | -0.06 | 0.04 | -1.64 | 1008.00 | .101 | 1.16 |
| Consonant Source  (dummy-coded) | -0.01 | 0.06 | -0.15 | 2016.00 | .877 | 1.33 |
| Democrat-Favoring Stimuli  (Dummy-coded) | 0.33 | 0.08 | 3.97 | 1008.00 | <.001^***^ | 1.13 |
| Partisan Extremity | 0.12 | 0.04 | 3.14 | 1008.00 | .002^**^ | 1.18 |
| Political Interest | 0.03 | 0.03 | 1.00 | 1008.00 | .315 | 1.65 |
| Male  (dummy-coded) | 0.11 | 0.08 | 1.31 | 1008.00 | .189 | 1.15 |
| Age | -0.04 | 0.03 | -1.37 | 1008.00 | .170 | 1.23 |
| Education | 0.02 | 0.04 | 0.60 | 1008.00 | .548 | 1.26 |
| Trust in Media | 0.12 | 0.03 | 3.83 | 1008.00 | <.001^***^ | 1.84 |
| Trust in Institutions | 0.14 | 0.04 | 3.53 | 1008.00 | <.00^***^1 | 1.88 |
| Faith in Intuition for Facts | 0.06 | 0.04 | 1.59 | 1008.00 | .112 | 1.40 |
| Need for Evidence | 0.04 | 0.04 | 0.85 | 1008.00 | .398 | 1.53 |
| Truth is Political | 0.05 | 0.03 | 1.60 | 1008.00 | .109 | 1.46 |
| Conspiracy Beliefs | 0.47 | 0.06 | 8.25 | 1008.00 | <.001^***^ | 1.66 |
| News Exposure | 0.05 | 0.03 | 1.52 | 1008.00 | .128 | 1.83 |

| Table 3: Perceived Recognition of Real News Posts | | | | | | |
| --- | --- | --- | --- | --- | --- | --- |
|  | Est. | S.E. | t val. | d.f. | p | VIF |
| (Intercept) | -1.06 | 0.32 | -3.27 | 1544.31 | .001^**^ |  |
| Alternative Outlet  (dummy-coded) | -0.11 | 0.05 | -2.36 | 4190.42 | .018^*^ | 1.13 |
| NML | -0.17 | 0.06 | -2.76 | 1398.37 | .006^**^ | 1.60 |
| Information Literacy | -0.04 | 0.03 | -1.32 | 1397.96 | .186 | 1.13 |
| Consonant Outlet  (dummy-coded) | 0.03 | 0.05 | 0.64 | 4190.46 | .524 | 1.13 |
| Democrat-Favoring Stimuli  (dummy-coded) | 0.03 | 0.08 | 0.33 | 1397.64 | .745 | 1.20 |
| Partisan Extremity | 0.12 | 0.03 | 3.55 | 1396.54 | <.001^***^ | 1.19 |
| Political Interest | 0.23 | 0.04 | 6.42 | 1396.58 | <.001^***^ | 1.61 |
| Male  (dummy-coded) | 0.35 | 0.08 | 4.61 | 1397.05 | <.001^***^ | 1.14 |
| Non-Binary  (dummy-coded) | -0.55 | 0.51 | -1.08 | 1396.41 | .282 | 1.02 |
| Age | 0.14 | 0.02 | 6.17 | 1397.43 | <.001^***^ | 1.36 |
| Education | 0.00 | 0.03 | -0.12 | 1397.03 | .901 | 1.20 |
| Trust in Media | -0.06 | 0.03 | -1.95 | 1396.96 | .051 | 2.11 |
| Trust in Institutions | 0.29 | 0.04 | 6.94 | 1396.59 | <.001^***^ | 2.29 |
| Faith in Intuition for Facts | -0.06 | 0.04 | -1.64 | 1396.72 | .101 | 1.37 |
| Need for Evidence | 0.04 | 0.04 | 0.90 | 1396.57 | .366 | 1.67 |
| Truth is Political | 0.13 | 0.03 | 4.47 | 1396.46 | <.001^***^ | 1.42 |
| Conspiracy Beliefs | 0.44 | 0.05 | 8.39 | 1399.16 | <.001^***^ | 1.57 |
| Attention Check Passed | 0.17 | 0.08 | 2.08 | 4665.25 | .038^*^ | 1.01 |
| Asked about Others’ Impressions (dummy-coded) | 0.17 | 0.08 | 2.21 | 1396.75 | .027^*^ | 1.01 |
| News Exposure | 0.16 | 0.03 | 6.06 | 1396.73 | <.001^***^ | 1.47 |

| Table 4: Perceived Accuracy of Real News Posts | | | | | | |
| --- | --- | --- | --- | --- | --- | --- |
|  | Est. | S.E. | t val. | d.f. | p | VIF |
| (Intercept) | 0.72 | 0.26 | 2.80 | 1555.02 | .005^**^ |  |
| Alternative Outlet (dummy-coded) | -0.09 | 0.04 | -2.32 | 4190.93 | .020^*^ | 1.13 |
| NML | 0.11 | 0.05 | 2.19 | 1399.00 | .029^*^ | 1.60 |
| Information Literacy | 0.04 | 0.03 | 1.45 | 1398.56 | .146 | 1.13 |
| Consonant Outlet (dummy-coded) | 0.04 | 0.04 | 0.94 | 4190.96 | .348 | 1.13 |
| Democrat-Favoring Stimuli (dummy-coded) | -0.45 | 0.06 | -7.27 | 1398.22 | <.001^***^ | 1.20 |
| Partisan Extremity | 0.18 | 0.03 | 6.77 | 1397.05 | <.001^***^ | 1.19 |
| Political Interest | 0.15 | 0.03 | 5.30 | 1397.09 | <.001^***^ | 1.61 |
| Male (dummy-coded) | 0.14 | 0.06 | 2.34 | 1397.59 | .020^*^ | 1.14 |
| Non-Binary (dummy-coded) | 0.26 | 0.40 | 0.64 | 1396.91 | .522 | 1.02 |
| Age | 0.07 | 0.02 | 3.65 | 1398.00 | <.001^***^ | 1.36 |
| Education | -0.07 | 0.03 | -2.53 | 1397.58 | .012^*^ | 1.20 |
| Trust in Media | -0.03 | 0.03 | -1.10 | 1397.50 | .272 | 2.11 |
| Trust in Institutions | 0.16 | 0.03 | 5.00 | 1397.10 | <.001^***^ | 2.29 |
| Faith in Intuition for Facts | 0.06 | 0.03 | 2.30 | 1397.24 | .022^*^ | 1.37 |
| Need for Evidence | 0.11 | 0.03 | 3.52 | 1397.08 | <.001^***^ | 1.67 |
| Truth is Political | 0.02 | 0.02 | 0.79 | 1396.97 | .429 | 1.42 |
| Conspiracy Beliefs | 0.26 | 0.04 | 6.34 | 1399.85 | <.001^***^ | 1.57 |
| Attention Check Passed | 0.11 | 0.07 | 1.61 | 4698.69 | .107 | 1.01 |
| Asked about Others’ Impressions (dummy-coded) | -0.01 | 0.06 | -0.23 | 1397.28 | .819 | 1.01 |
| News Exposure | 0.03 | 0.02 | 1.62 | 1397.25 | .106 | 1.47 |

| Table 5: Perceived Recognition of Outlets | | | | | | |
| --- | --- | --- | --- | --- | --- | --- |
|  | Est. | S.E. | t val. | d.f. | p | VIF |
| (Intercept) | 1.18 | 0.26 | 4.50 | 1606.56 | <.001^***^ |  |
| Alternative Outlet (dummy-coded) | -1.20 | 0.05 | -25.52 | 4190.76 | <.001^***^ | 1.13 |
| NML | 0.39 | 0.05 | 7.85 | 1399.50 | <.001^***^ | 1.60 |
| Information Literacy | 0.10 | 0.03 | 3.89 | 1398.92 | <.001^***^ | 1.13 |
| Consonant Outlet (dummy-coded) | 0.30 | 0.05 | 6.39 | 4190.81 | <.001^***^ | 1.13 |
| Democrat-Favoring Stimuli (dummy-coded) | 0.03 | 0.06 | 0.46 | 1398.47 | .649 | 1.20 |
| Partisan Extremity | 0.03 | 0.03 | 0.94 | 1396.93 | .345 | 1.19 |
| Political Interest | 0.09 | 0.03 | 3.12 | 1396.98 | .002^**^ | 1.61 |
| Male (dummy-coded) | -0.05 | 0.06 | -0.77 | 1397.64 | .439 | 1.14 |
| Non-Binary (dummy-coded) | -0.31 | 0.41 | -0.76 | 1396.74 | .446 | 1.02 |
| Age | -0.02 | 0.02 | -0.84 | 1398.18 | .403 | 1.36 |
| Education | 0.00 | 0.03 | -0.10 | 1397.62 | .917 | 1.20 |
| Trust in Media | -0.07 | 0.03 | -2.75 | 1397.52 | .006^**^ | 2.11 |
| Trust in Institutions | 0.25 | 0.03 | 7.69 | 1396.99 | <.001^***^ | 2.29 |
| Faith in Intuition for Facts | 0.04 | 0.03 | 1.51 | 1397.17 | .132 | 1.37 |
| Need for Evidence | 0.06 | 0.03 | 1.86 | 1396.96 | .064 | 1.67 |
| Truth is Political | -0.04 | 0.02 | -1.78 | 1396.82 | .075 | 1.42 |
| Conspiracy Beliefs | 0.01 | 0.04 | 0.34 | 1400.61 | .732 | 1.57 |
| Attention Checks Passed | 0.02 | 0.08 | 0.29 | 4866.00 | .768 | 1.01 |
| Asked about Others’ Impressions (dummy-coded) | 0.08 | 0.06 | 1.29 | 1397.22 | .198 | 1.01 |
| News Exposure | 0.15 | 0.02 | 7.26 | 1397.19 | <.001^***^ | 1.47 |

| Table 6: Perceived Accuracy of Outlets | | | | | | |
| --- | --- | --- | --- | --- | --- | --- |
|  | Est. | S.E. | t val. | d.f. | P | VIF |
| (Intercept) | 1.23 | 0.23 | 5.39 | 1670.09 | <.001^***^ |  |
| Alternative Outlet (dummy-coded) | -0.10 | 0.05 | -2.15 | 4190.71 | .031^*^ | 1.13 |
| NML | 0.14 | 0.04 | 3.29 | 1400.26 | .001^**^ | 1.60 |
| Information Literacy | 0.05 | 0.02 | 1.97 | 1399.51 | .049^*^ | 1.13 |
| Consonant Outlet (dummy-coded) | 0.65 | 0.05 | 13.85 | 4190.78 | <.001^***^ | 1.13 |
| Democrat-Favoring Stimuli (dummy-coded) | -0.02 | 0.05 | -0.39 | 1398.93 | .697 | 1.20 |
| Partisan Extremity | 0.01 | 0.02 | 0.60 | 1396.93 | .551 | 1.19 |
| Political Interest | 0.02 | 0.02 | 0.63 | 1397.00 | .531 | 1.61 |
| Male (dummy-coded) | -0.13 | 0.05 | -2.54 | 1397.85 | .011^*^ | 1.14 |
| Non-Binary (dummy-coded) | -0.43 | 0.35 | -1.23 | 1396.69 | .220 | 1.02 |
| Age | 0.00 | 0.02 | -0.31 | 1398.55 | .758 | 1.36 |
| Education | -0.06 | 0.02 | -2.49 | 1397.83 | .013^*^ | 1.20 |
| Trust in Media | 0.19 | 0.02 | 8.47 | 1397.70 | <.001^***^ | 2.11 |
| Trust in Institutions | 0.30 | 0.03 | 10.52 | 1397.02 | <.001^***^ | 2.29 |
| Faith in Intuition for Facts | 0.04 | 0.02 | 1.56 | 1397.25 | .120 | 1.37 |
| Need for Evidence | 0.02 | 0.03 | 0.71 | 1396.98 | .478 | 1.67 |
| Truth is Political | -0.02 | 0.02 | -0.96 | 1396.79 | .339 | 1.42 |
| Conspiracy Beliefs | 0.09 | 0.04 | 2.45 | 1401.71 | .015^*^ | 1.57 |
| Attention Checks Passed | 0.00 | 0.08 | -0.01 | 5067.13 | .992 | 1.02 |
| Asked about Others’ Impressions (dummy-coded) | -0.01 | 0.05 | -0.10 | 1397.31 | .918 | 1.01 |
| News Exposure | 0.09 | 0.02 | 5.15 | 1397.27 | <.001^***^ | 1.47 |

**Appendix G**

**Simple Slopes Analyses Per Significant Interactions**

**Study 1**

Perceived Accuracy ~ NML*Partisan Extremity*Alternative Outlet (dummy-coded) + (1|ID)

While Alternative Outlet (dummy-coded)(2nd moderator) = 0.000 (0)

SIMPLE SLOPES ANALYSIS

Slope of NML when Partisan Extremity = 3.000:

Est. S.E. t val. p

------- ------- -------- -------

0.422 0.094 4.507 0.000

Slope of NML when Partisan Extremity = 2.000:

Est. S.E. t val. p

------- ------- -------- -------

0.261 0.068 3.815 0.000

Slope of NML when Partisan Extremity = 1.000:

Est. S.E. t val. p

------- ------- -------- -------

0.099 0.084 1.182 0.238

Slope of NML when Partisan Extremity = 0.000:

Est. S.E. t val. p

-------- ------- -------- -------

-0.062 0.126 -0.495 0.621

While Alternative Outlet (dummy-coded)(2nd moderator) = 1.000 (1)

SIMPLE SLOPES ANALYSIS

Slope of NML when Partisan Extremity = 3.000:

Est. S.E. t val. p

------- ------- -------- -------

0.468 0.112 4.161 0.000

Slope of NML when Partisan Extremity = 2.000:

Est. S.E. t val. p

------- ------- -------- -------

0.109 0.082 1.334 0.182

Slope of NML when Partisan Extremity = 1.000:

Est. S.E. t val. p

-------- ------- -------- -------

-0.249 0.101 -2.474 0.013

Slope of NML when Partisan Extremity = 0.000:

Est. S.E. t val. p

-------- ------- -------- -------

-0.607 0.151 -4.017 0.000

**Study 2**

Perceived Accuracy (Post-Level) ~ NML*Partisan Extremity*Alternative Outlet (dummy-coded) + (1|ID)

While Alternative Outlet (dummy-coded)(2nd moderator) = 0.000 (0)

SIMPLE SLOPES ANALYSIS

Slope of NML when Partisan Extremity = 3.000:

Est. S.E. t val. p

------- ------- -------- -------

0.368 0.066 5.577 0.000

Slope of NML when Partisan Extremity = 2.000:

Est. S.E. t val. p

------- ------- -------- -------

0.372 0.046 8.064 0.000

Slope of NML when Partisan Extremity = 1.000:

Est. S.E. t val. p

------- ------- -------- -------

0.376 0.051 7.402 0.000

Slope of NML when Partisan Extremity = 0.000:

Est. S.E. t val. p

------- ------- -------- -------

0.380 0.076 5.031 0.000

While Alternative Outlet (dummy-coded)(2nd moderator) = 1.000 (1)

SIMPLE SLOPES ANALYSIS

Slope of NML when Partisan Extremity = 3.000:

Est. S.E. t val. p

------- ------- -------- -------

0.483 0.085 5.696 0.000

Slope of NML when Partisan Extremity = 2.000:

Est. S.E. t val. p

------- ------- -------- -------

0.380 0.059 6.409 0.000

Slope of NML when Partisan Extremity = 1.000:

Est. S.E. t val. p

------- ------- -------- -------

0.277 0.065 4.244 0.000

Slope of NML when Partisan Extremity = 0.000:

Est. S.E. t val. p

------- ------- -------- -------

0.174 0.097 1.794 0.073

Perceived Recognition (Outlet-Level) ~ NML*Partisan Extremity*Alternative Outlet (dummy-coded) + (1|ID)

While Alternative Outlet (dummy-coded)(2nd moderator) = 0.000 (0)

SIMPLE SLOPES ANALYSIS

Slope of NML when Partisan Extremity = 3.000:

Est. S.E. t val. p

------- ------- -------- -------

0.759 0.067 11.274 0.000

Slope of NML when Partisan Extremity = 2.000:

Est. S.E. t val. p

------- ------- -------- -------

0.771 0.047 16.357 0.000

Slope of NML when Partisan Extremity = 1.000:

Est. S.E. t val. p

------- ------- -------- -------

0.782 0.052 15.065 0.000

Slope of NML when Partisan Extremity = 0.000:

Est. S.E. t val. p

------- ------- -------- -------

0.793 0.077 10.274 0.000

While Alternative Outlet (dummy-coded)(2nd moderator) = 1.000 (1)

SIMPLE SLOPES ANALYSIS

Slope of NML when Partisan Extremity = 3.000:

Est. S.E. t val. p

------- ------- -------- -------

0.093 0.092 1.017 0.309

Slope of NML when Partisan Extremity = 2.000:

Est. S.E. t val. p

-------- ------- -------- -------

-0.013 0.064 -0.196 0.845

Slope of NML when Partisan Extremity = 1.000:

Est. S.E. t val. p

-------- ------- -------- -------

-0.118 0.071 -1.676 0.094

Slope of NML when Partisan Extremity = 0.000:

Est. S.E. t val. p

-------- ------- -------- -------

-0.224 0.105 -2.134 0.033

**Appendix H**

**Predicting Sharing Likelihood**

**Study 1: Fake News Posts**

***Methods***

**Sharing Likelihood.** Following Pennycook and Rand (2019), participants rated their agreement with the statement, “I would likely share this post on social media or messaging apps” on a 7-point scale from "strongly disagree" to "strongly agree.” By outlet, participants rated consonant posts from the alternative outlet (Just the News) as *M* = 2.92 (*SD* = 2.11), the dissonant (CNN or Fox) outlet as *M* = 2.91 (*SD* = 2.09), and the consonant (CNN or Fox) outlet as *M* = 2.95 (*SD* = 2.12).

***Results***

| Table 1: Sharing Likelihood of Fake News Content | | | | | | |
| --- | --- | --- | --- | --- | --- | --- |
|  | Est. | S.E. | t val. | d.f. | p | VIF |
| (Intercept) | 0.54 | 0.33 | 1.65 | 1009.47 | .100 |  |
| Perceived Recognition | 0.14 | 0.02 | 8.84 | 2972.80 | < .001^***^ | 1.47 |
| Perceived Accuracy | 0.35 | 0.02 | 21.93 | 2904.34 | < .001^***^ | 1.37 |
| Alternative Outlet (dummy-coded) | 0.08 | 0.05 | 1.62 | 2003.08 | .105 | 1.34 |
| NML | -0.16 | 0.06 | -2.54 | 993.25 | .011^*^ | 1.54 |
| Information Literacy | -0.12 | 0.04 | -3.51 | 994.18 | < .001^***^ | 1.16 |
| Consonant Outlet (dummy-coded) | 0.03 | 0.05 | 0.70 | 1999.95 | .482 | 1.33 |
| Democrat-Favoring Stimuli (dummy-coded) | -0.33 | 0.08 | -4.18 | 1004.00 | < .001^***^ | 1.14 |
| Partisan Extremity | 0.10 | 0.04 | 2.92 | 997.15 | .004^**^ | 1.19 |
| Political Interest | 0.08 | 0.03 | 2.60 | 993.90 | .009^**^ | 1.65 |
| Male (dummy-coded) | -0.23 | 0.08 | -2.90 | 994.09 | .004^**^ | 1.16 |
| Age | -0.14 | 0.03 | -5.45 | 994.37 | < .001^***^ | 1.23 |
| Education | -0.02 | 0.04 | -0.64 | 994.12 | .520 | 1.26 |
| Trust in Media | 0.10 | 0.03 | 3.37 | 1000.49 | .001^**^ | 1.85 |
| Trust in Institutions | 0.10 | 0.04 | 2.62 | 996.34 | .009^**^ | 1.89 |
| Faith in Intuition for Facts | -0.05 | 0.04 | -1.20 | 993.03 | .231 | 1.4 |
| Need for Evidence | -0.07 | 0.04 | -1.61 | 992.41 | .109 | 1.53 |
| Truth is Political | 0.09 | 0.03 | 3.07 | 994.20 | .002^**^ | 1.46 |
| Conspiracy Beliefs | 0.26 | 0.06 | 4.79 | 1021.89 | < .001 | 1.69 |
| News Exposure | 0.16 | 0.03 | 5.45 | 1008.53 | < .001 | 1.85 |

**Study 2: Real News Posts**

***Methods***

**Sharing Likelihood by Post.** Participants rated their agreement with the statement, “I would likely share this post on social media or messaging apps” on a 7-point scale from "strongly disagree" to "strongly agree.” Descriptive statistics for posts by outlet were as follows: alternative outlet (*M* = 3.10, *SD* = 1.99), mainstream outlet (*M* = 3.11, *SD* = 1.99), the dissonant outlet (*M* = 3.07, *SD* = 2.00), and the consonant outlet (*M* = 3.16, *SD* = 1.97).

**Sharing Likelihood by Outlet.** Participants rated their agreement with the statement, “I would likely share stories from this outlet on social media or messaging apps” on a 7-point scale from "strongly disagree" to "strongly agree.” Descriptive statistics by outlet were as follows: alternative outlet (*M* = 3.28, *SD* = 1.84), mainstream outlet (*M* = 3.52, *SD* = 1.93), the dissonant outlet (*M* = 3.07, *SD* = 1.99), and the consonant outlet (*M* = 3.76, *SD* = 1.98).

***Results***

| Table 2: Sharing Likelihood of Real News Posts | | | | | | |
| --- | --- | --- | --- | --- | --- | --- |
|  | Est. | S.E. | t val. | d.f. | p | VIF |
| (Intercept) | 0.01 | 0.28 | 0.05 | 1485.56 | .960 |  |
| Perceived Recognition | 0.13 | 0.01 | 12.00 | 5393.90 | < .001^***^ | 1.34 |
| Perceived Accuracy | 0.31 | 0.01 | 23.69 | 5345.20 | < .001^***^ | 1.32 |
| Alternative Outlet (dummy-coded) | 0.06 | 0.03 | 1.81 | 4180.26 | .071 | 1.13 |
| NML | -0.34 | 0.05 | -6.43 | 1391.37 | < .001^***^ | 1.61 |
| Information Literacy | -0.08 | 0.03 | -2.84 | 1387.06 | .005^**^ | 1.13 |
| Consonant Outlet (dummy-coded) | 0.06 | 0.03 | 1.84 | 4178.47 | .066 | 1.13 |
| Democrat-favoring Stimuli (dummy-coded) | -0.33 | 0.07 | -4.79 | 1402.91 | < .001^***^ | 1.21 |
| Partisan Extremity | 0.13 | 0.03 | 4.55 | 1396.57 | < .001^***^ | 1.20 |
| Political Interest | 0.16 | 0.03 | 5.19 | 1397.70 | < .001^***^ | 1.62 |
| Male | 0.19 | 0.07 | 2.80 | 1390.57 | .005^**^ | 1.14 |
| Non-Binary | -0.06 | 0.44 | -0.14 | 1384.99 | .886 | 1.02 |
| Age | -0.09 | 0.02 | -4.59 | 1395.71 | < .001^***^ | 1.37 |
| Education | -0.09 | 0.03 | -2.95 | 1386.63 | .003^**^ | 1.20 |
| Trust in Media | 0.03 | 0.03 | 1.05 | 1385.68 | .294 | 2.11 |
| Trust in Institutions | 0.24 | 0.04 | 6.57 | 1398.89 | < .001^***^ | 2.3 |
| Faith in Intuition for Facts | 0.11 | 0.03 | 3.59 | 1388.18 | < .001^***^ | 1.37 |
| Need for Evidence | -0.09 | 0.04 | -2.69 | 1387.74 | .007^**^ | 1.67 |
| Truth is Political | 0.08 | 0.03 | 3.11 | 1390.31 | .002^**^ | 1.42 |
| Conspiracy Beliefs | 0.39 | 0.05 | 8.52 | 1407.66 | < .001^***^ | 1.58 |
| Attention Checks Passed | -0.04 | 0.05 | -0.72 | 4478.39 | .471 | 1.01 |
| Distractor (other’s impressions) (dummy-coded) | 0.12 | 0.07 | 1.86 | 1386.30 | .063 | 1.01 |
| News Exposure | 0.11 | 0.02 | 4.93 | 1395.03 | < .001^***^ | 1.48 |

| Table 2: Sharing Likelihood by Outlet | | | | | | |
| --- | --- | --- | --- | --- | --- | --- |
|  | Est. | S.E. | t val. | d.f. | p | VIF |
| (Intercept) | 0.46 | 0.29 | 1.60 | 1483.24 | .110 |  |
| Perceived Recognition | 0.09 | 0.01 | 7.72 | 5234.95 | < .001^***^ | 1.56 |
| Perceived Accuracy | 0.51 | 0.01 | 45.57 | 4996.66 | < .001^***^ | 1.41 |
| Alternative Outlet (dummy-coded) | 0.14 | 0.04 | 4.14 | 4345.03 | < .001^***^ | 1.30 |
| NML | -0.41 | 0.05 | -8.67 | 1410.03 | < .001^***^ | 1.62 |
| Information Literacy | -0.06 | 0.03 | -2.42 | 1398.02 | .016^*^ | 1.13 |
| Consonant Outlet (dummy-coded) | 0.10 | 0.03 | 3.11 | 4224.19 | .002^**^ | 1.17 |
| Democrat-favoring Stimuli (dummy-coded) | -0.06 | 0.10 | -0.62 | 1393.16 | .537 | 3.38 |
| Partisan Extremity | 0.01 | 0.02 | 0.57 | 1393.15 | .570 | 3.59 |
| Political Interest | 0.09 | 0.03 | 3.38 | 1393.21 | .001^**^ | 1.19 |
| Male | 0.09 | 0.03 | 3.33 | 1395.75 | .001^**^ | 1.61 |
| Non-Binary | 0.11 | 0.06 | 1.84 | 1394.70 | .066 | 1.14 |
| Age | -0.15 | 0.39 | -0.39 | 1393.20 | .695 | 1.02 |
| Education | -0.09 | 0.02 | -5.39 | 1393.85 | < .001^***^ | 1.37 |
| Trust in Media | -0.06 | 0.03 | -2.25 | 1394.92 | .025^*^ | 1.20 |
| Trust in Institutions | 0.03 | 0.03 | 1.05 | 1419.91 | .292 | 2.18 |
| Faith in Intuition for Facts | 0.17 | 0.03 | 5.54 | 1418.92 | < .001^***^ | 2.31 |
| Need for Evidence | 0.10 | 0.03 | 3.84 | 1393.92 | < .001^***^ | 1.37 |
| Truth is Political | -0.12 | 0.03 | -3.84 | 1393.92 | < .001^***^ | 1.67 |
| Conspiracy Beliefs | 0.08 | 0.02 | 3.41 | 1393.73 | .001^**^ | 1.42 |
| Attention Checks Passed | 0.26 | 0.04 | 6.47 | 1396.50 | < .001^***^ | 1.57 |
| Distractor (other’s impressions) (dummy-coded) | 0.06 | 0.05 | 1.18 | 4568.73 | .24 | 1.01 |
| News Exposure | 0.11 | 0.06 | 1.98 | 1393.73 | .048^*^ | 1.01 |
